# RiboMiner: a toolset for mining multi-dimensional features of the translatome with ribosome profiling data

**DOI:** 10.1186/s12859-020-03670-8

**Published:** 2020-08-01

**Authors:** Fajin Li, Xudong Xing, Zhengtao Xiao, Gang Xu, Xuerui Yang

**Affiliations:** 1 MOE Key Laboratory of Bioinformatics, School of Life Sciences, Tsinghua University, Medical Science Building D231, Beijing, 100084 China; 2 Center for Synthetic & Systems Biology, Tsinghua University, Beijing, 100084 China; 3 Joint Graduate Program of Peking-Tsinghua-National Institute of Biological Science, Tsinghua University, Beijing, 100084 China

## Abstract

**Background:**

Ribosome profiling has been widely used for studies of translation under a large variety of cellular and physiological contexts. Many of these studies have greatly benefitted from a series of data-mining tools designed for dissection of the translatome from different aspects. However, as the studies of translation advance quickly, the current toolbox still falls in short, and more specialized tools are in urgent need for deeper and more efficient mining of the important and new features of the translation landscapes.

**Results:**

Here, we present RiboMiner, a bioinformatics toolset for mining of multi-dimensional features of the translatome with ribosome profiling data. RiboMiner performs extensive quality assessment of the data and integrates a spectrum of tools for various metagene analyses of the ribosome footprints and for detailed analyses of multiple features related to translation regulation. Visualizations of all the results are available. Many of these analyses have not been provided by previous methods. RiboMiner is highly flexible, as the pipeline could be easily adapted and customized for different scopes and targets of the studies.

**Conclusions:**

Applications of RiboMiner on two published datasets did not only reproduced the main results reported before, but also generated novel insights into the translation regulation processes. Therefore, being complementary to the current tools, RiboMiner could be a valuable resource for dissections of the translation landscapes and the translation regulations by mining the ribosome profiling data more comprehensively and with higher resolution. RiboMiner is freely available at https://github.com/xryanglab/RiboMiner and https://pypi.org/project/RiboMiner.

## Background

Based on deep sequencing of the ribosome-protected mRNA fragments, ribosome profiling enables genome-wide investigations of translation with sub-codon resolution [1]. In the past decade, ribosome profiling has been widely used for studies of translation under various contexts in human and almost all the major model organisms such as bacteria, *C. elegans*, yeast, and mouse, et al. [2, 3]. In these studies, the most popular applications of ribosome profiling data include quantifications of translation efficiency (TE) [4, 5], annotations of open reading frames (ORFs) [4, 6], meta-gene analysis of the ribosome distribution patterns [7, 8], and identification of translation initiation sites and pausing regions [3, 7].

**Fig. 1:**
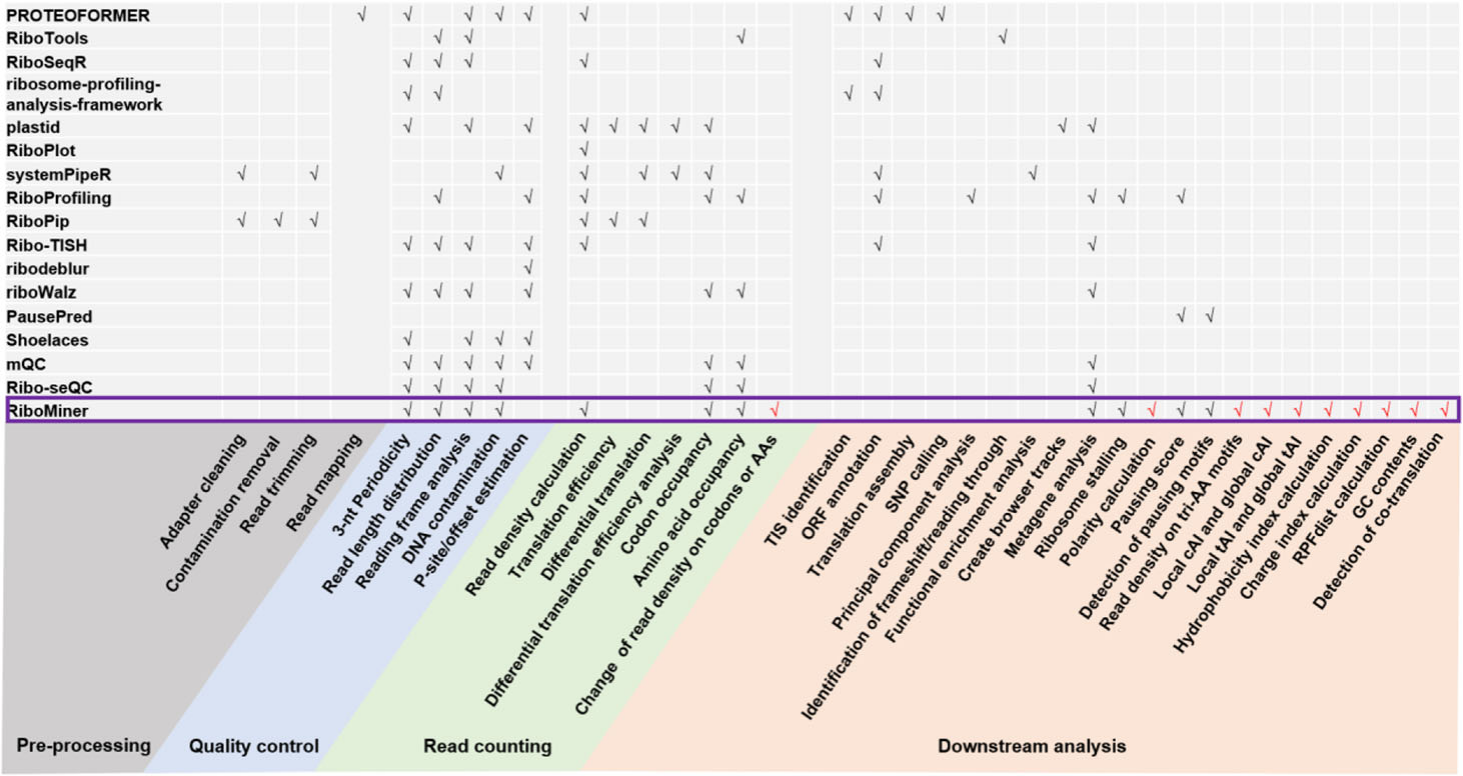
Major functions of the data analysis tools for ribosome profiling. Partly adapted from a review article by Wang, et al. [9]. RiboMiner is highlighted at the bottom, and the red ticks represent the unique functions of RiboMiner

A large variety of algorithms, software, and online resources have been developed to accommodate the ever-growing needs for data processing and analysis of ribosome profiling as well as presentation and interpretation of the results [9–11]. For example, RiboCode [12], ORF-RATER [13], Ribowave [14], ORFscore [15], RiboTaper [16], and Ribo-TISH [17] were designed for annotation of active ORFs. Xtail [18], anota [19], Riborex [20], Babel [21], and RiboDiff [22] were developed for quantification of TE changes. In addition to these highly focused analyses, more customized down-stream analyses of ribosome profiling data are of great value as well for better understanding of the translatome and the translation regulation. As summarized in Fig. 1, these pipelines implemented a broad range of data analyses, from pre-processing to down-stream information mining [17, 23–36]. For example, RiboPip is focused on the pre-processing of ribosome profiling and RNA-seq data; mQC [35] and Ribo-seQC [36] were mainly designed for quality control of the data; RiboPlot [28] and Shoelaces [34] can be used for quality control and visualization of the data; PausePred [33] was specifically designed to identify pausing motifs; RiboTools [24] is a Galaxy toolbox with functions including detection of translational ambiguities and identification of readthrough events; PROTEOFORMER [23] is a python package for automatic processing of ribosome profiling data, which includes SNP calling, ORF assembly and TIS identification; Plastid [27] is used for differential translation analysis and metagene analysis; RiboProfiling [30], riboSeqR [25], and systemPipeR [29] are R packages for tasks such as data pre-processing, quality control, TE calculation and ORF annotation, et al. These tools together cover many key procedures for ribosome profiling data analysis and presentation. However, as shown in Fig. 1, new types of analyses, many of which are related to the complicated machinery of translation regulation and only emerged in recent studies, have not been implemented by the current methods.

Here, we present RiboMiner, a python toolset for ribosome profiling data analysis, in response to the emerging needs for deeper mining of the hidden information about translation and its regulation, such as co-translation events, metagene analyses of the ribosome footprint density, and the potential regulatory factors of ribosome distribution, e.g., codon usage, tRNA gene copy numbers, and properties of nascent amino acids (Fig. 1). Characterization of these multi-dimensional features, under specific experimental or physiological conditions, provides closer views on the shift of translation landscapes with more details, which could be informative for pursuing the underlying machinery of translation regulation.

## Implementation

### Overview of RiboMiner

**Fig. 2:**
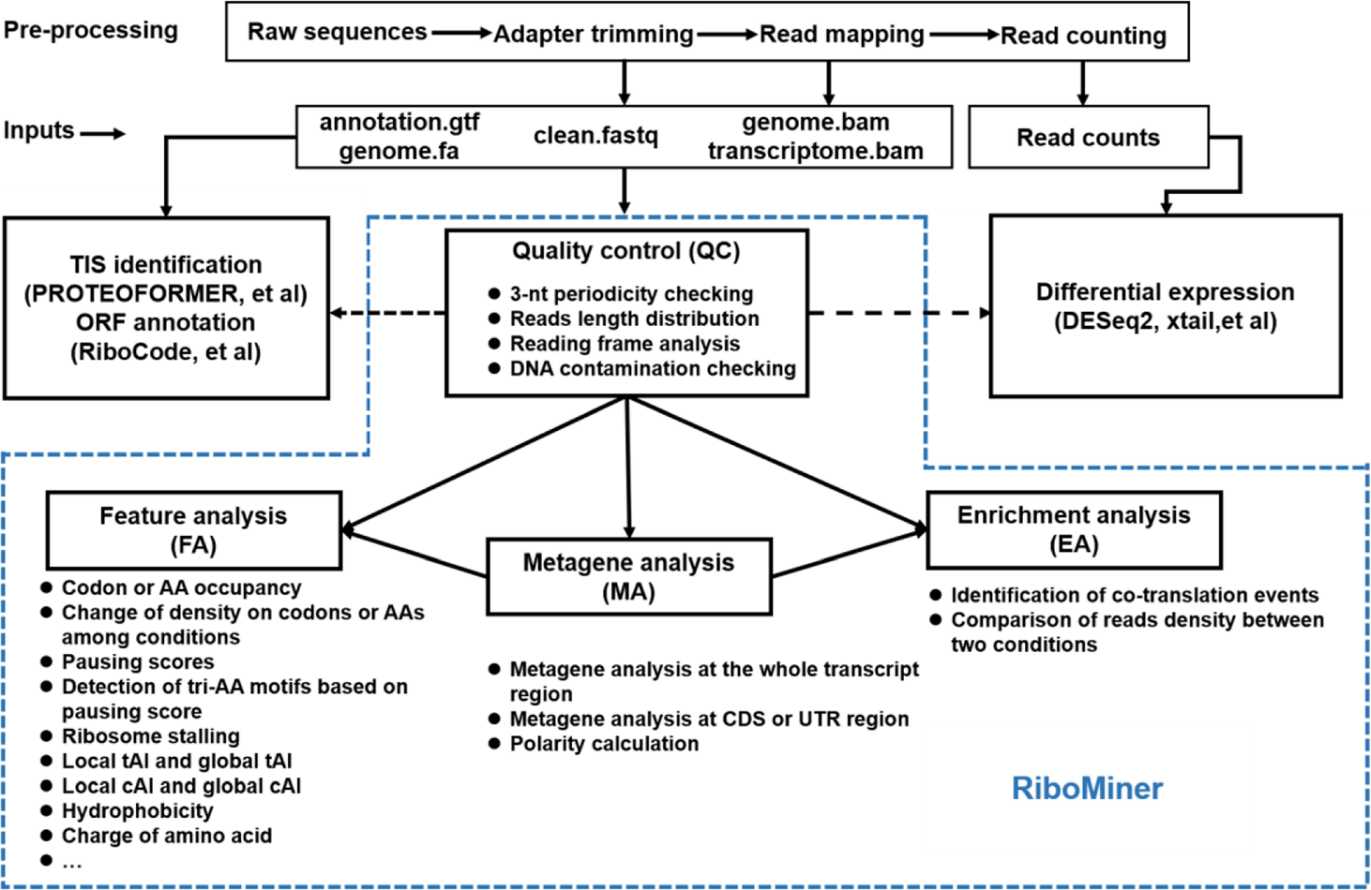
Overview of RiboMiner. There are four function modules in RiboMiner. QC: quality control of ribosome profiling data. MA: metagene analysis for different samples with the full genome or pre-defined gene sets. FA: feature analysis for given sets of genes, which covers a series of translation-related indices. EA: enrichment analysis for the data of selective ribosome profiling to identify the potential co-translation regulation events

The pipeline of RiboMiner is composed of 4 major function modules (Fig. 2): 1) Quality Control (QC), designed for assessment of ribosome profiling data quality with a multitude of benchmarks including 3-nt periodicity, distribution of read lengths, reads distribution in non-coding genome/transcriptome, etc. 2) Metagene Analysis (MA) for global distribution patterns of ribosome footprints on pooled transcripts of the full or any given subset of the transcriptome. This analysis helps identifying potential ribosome stalling events that take place at the global scale or just for subsets of the transcripts. Via direct comparisons between different conditions, metagene analysis of the footprints shed lights on how and at which stage these conditions perturb translation. 3) Feature Analysis (FA) for mining of various features that are enriched in predefined gene sets, e.g., the subset of genes with ribosome stalling events detected by MA as introduced above. The features being tested here include ribosome footprint densities on different amino acid (AA) and tri-amino acid (tri-AA) motifs, local and global codon adaptation index, local and global tRNA adaptation index, AA hydrophobicity and charge, etc. By identifying such features that are enriched in selected genes, this module of functions could provide insights into the machinery of translation landscape shift, from a multitude of different perspectives. 4) Enrichment Analysis (EA), designed for direct comparison of the ribosome footprint distributions across two experiments. For instance, this function can be used for data analysis of selective ribosome profiling to help the identification of co-translation regulation.

The python package of RiboMiner is freely available at https://github.com/xryanglab/RiboMiner and https://pypi.org/project/RiboMiner. We also offer a Docker image for RiboMiner at https://hub.docker.com/r/yanglab/ribocode_ribominer. The RiboMiner pipeline, including the testing data used in the present study, is also available as a Gene Container Service (GCS) on the Huawei Cloud.

### Inputs of RiboMiner

As previously described, the common practice of raw data pre-processing before downstream analysis of ribosome profiling data includes quality control of sequencing, adapter trimming, read mapping and read counting (Fig. 2). The counts files could be used for differential expression analysis or quantification of differential translation efficiency with specialized tools such as DESeq2 [37] or Xtail [18]. The BAM files generated by read mapping can be used for TIS identification [23, 26] or ORF annotation [12, 13, 15] with specialized tools such as RiboCode [12] and PROTEOFORMER [23]. There are three categories of input files for RiboMiner (Fig. 2): 1) genome sequences in FASTA format and an annotation file in GTF format, which would be used for annotations of the RNA transcripts and the protein coding sequences; 2) RPF sequences in FASTA format, after pre-processing such as adapter trimming and quality filtering; 3) Two BAM files generated by mapping of the RPF reads to the genome and the transcriptome. These BAM files can be supplied by the users or generated by the script we have offered, which can be found on the GitHub page of RiboMiner. See supplementary file for a detailed tutorial of RiboMiner, including preparation of input files and all the down-stream analyses.

### Data preparation

Alternative splicing of the eukaryotic genes generates multiple transcript isoforms for each gene [38]. To avoid ambiguous alignments of the sequencing reads, the longest transcript of each protein coding gene would be used for the following analyses. The function *OutputTranscriptInfo* performs this task and generates annotation files containing all the selected transcripts of the protein coding genes. *GetProteinCodingSequence* returns the sequences of these transcripts, the protein coding sequences, and the amino acid sequences, whereas *GetUTRSequences* extracts the UTR sequences of these transcripts specifically. It should be noted that all the functions above are dependent on the transcript annotation file generated by *prepare_transcripts* of RiboCode [12] that our group developed before.

### Quality control

Four functions were designed for quality control of the ribosome footprints. *Periodicity*, which is adapted from *metaplots* of RiboCode [12], is used for assessment of the 3-nt periodicity and identification of P-sites of the ribosome footprints. It reports the distributions of RPFs aligned by their 5′ end in relative to the start and stop codons, which is done for the reads of each specific length or for all the reads combined. *RiboDensityOfDiffFrames* returns the read densities of each reading frame. *LengthDistribution* or *ReadsLengthOfSpecificRegions* provides the length distributions of all the ribosome footprints or the ones from specific regions such as CDS, 5’UTR, and 3’UTR. *StatisticReadsOnDNAsContam* counts the RPF reads mapped to introns and intergenic regions of the genome, which are potentially DNA contaminations or other non-ribosome-footprint fragments and could be indicative to the data quality in general.

### Metagene analysis

Metagene analysis aligns the transcripts of all the genes or a pre-defined gene set by their start codons and quantifies the relative read densities at each nucleotide or codon. Such analysis is particularly useful for identifying the potential global ribosome stalling sites under certain experimental or physiological conditions. *MetageneAnalysisForTheWholeRegions* can be used for calculating the read densities along the transcripts for an overall view, which would be helpful for testing whether the ribosome distributions are biasedly enriched. The function *PolarityCalculation* then evaluates the ribosome distribution bias for each gene and returns an overall distribution of such bias for all the genes. Finally, *MetageneAnalysis* was developed to zoom in and study the footprint densities in any particular region of the transcripts, including the UTR regions. This is particularly useful for allocating the ribosome stalling regions. All the results from the functions above can be readily presented as figures (see Supplementary file).

### Feature analysis

A number of factors have been shown to be involved in the regulation of translation initiation and elongation, such as poly-proline motifs [8, 39, 40], codon usage [41], tRNA gene copy numbers [42], amino acids with positive charges and high isoelectric points (pI) [43], etc. RiboMiner provides a series of functions for mining of such hidden features that are related to ribosome occupancy, with a goal of providing valuable insights into the molecular machinery of translation regulation. *RiboDensityAtEachKindAAOrCodon* calculates the ribosome footprint density at each amino acid (AA) or codon to show the differences under the experimental or physiological conditions. Furthermore, *PausingScore* was developed to quantify the ribosome density at each tri-AA motif and identify the motifs with enriched ribosome occupancy. Next, *RiboDensityAroundTripleteAAMotifs* can be used for computing the ribosome occupancy around the P/E site of the tri-AA motifs identified by the functions above.

tRNA adaptation index (tAI) and codon adaptation index (cAI) have been found to be potentially influential to translation, especially during elongation [41–43]. RiboMiner thereby provides two functions *tAI* and *cAI* to calculate the global tAI and cAI values as well as the local tAI and cAI values at each position along the transcripts for a specified organism. Note that although the weights of tAI are not exactly the same in all species, most of them are highly correlated to the weights fitted in yeast [44]. Finally, *hydropathyCharge* calculates the hydrophobicity and charge indexes of amino acids encoded by each codon along the transcripts.

Previously, the ratio of RPF read counts in 5’UTR to CDS was reported to be negatively correlated with the translation efficiency (TE) [45]. RiboMiner includes a function, *RPFdist*, to calculate these ratios, which would be potentially informative for evaluating the translation efficiencies in cases when the RNA-seq data in parallel with the ribosome profiling data is not available. See Supplementary file for more details about usages of these functions above.

### Enrichment analysis

Direct comparison between different ribosome profiling data could reveal the translation landscape shifts in details. For example, selective ribosome profiling (SeRP) is a powerful tool for studying the interaction of molecular chaperones and their potential targeting factors in the process of translation elongation [46]. It also reveals the co-translation events among different subunits of protein complexes [47]. Detailed analysis of the data from SeRP has been carefully done [46, 48], but these pipelines are not available in any of the current data analysis programs. Thus, RiboMiner incorporated a specially designed pipeline for mining of ribosome footprint enrichments with selective ribosome profiling data, which is quite different from the normal ribosome profiling data. This pipeline is composed of at least three steps: First, *RiboDensityAtEachPosition* calculates the ribosome density at each position for each transcript; Second, *EnrichmentAnalysis* performs an enrichment analysis by calculating the ribosome density ratio at each position in one ribosome profiling data over another one, e.g., ribosome profiling with IP of a specific protein and the normal ribosome profiling data. Finally, *PlotEnrichmentRatio* or *EnrichmentAnalysisForSingleTrans* generates plots of the results. In cases of multiple replicates for each condition, *enrichmentMeanDensity* can be used to calculate the mean ribosome density at each position ahead of the function *EnrichmentAnalysis*. It is worth noting that although this module was designed for SeRP data analysis, it could also be used for read density comparison between two normal ribosome profiling data under different conditions.

## Results

To showcase the application of RiboMiner for mining of insights into the translation regulation machineries, we used two published datasets, one from the study of eIF5A in translation regulation in yeast (GSE89704) [8] and the other from the study of co-translation of the aminoacyl-tRNA-synthetase complex (GSE116570) [47], which is specifically used for the Enrichment Analysis.

### Quality control

**Fig. 3:**
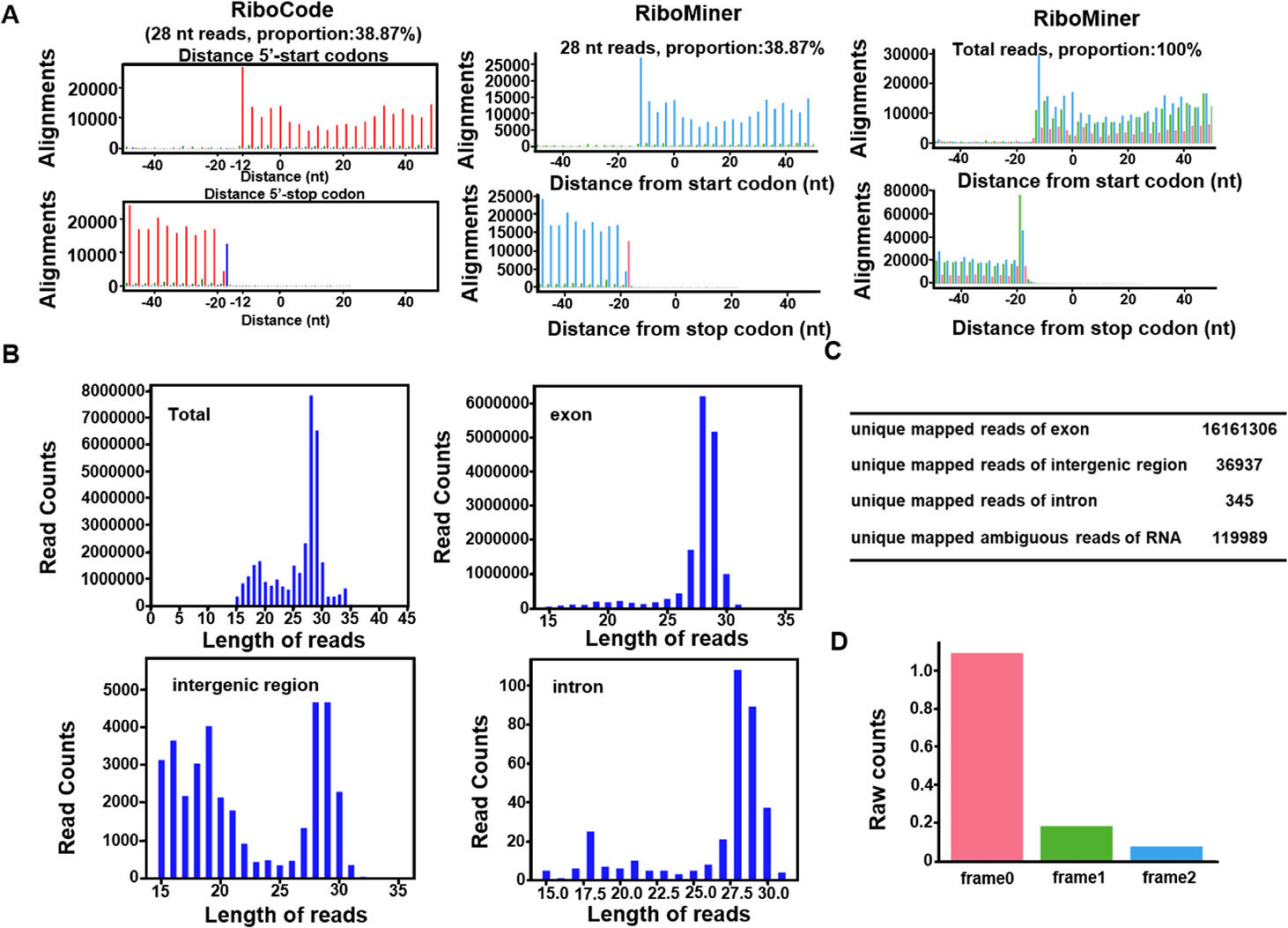
Results of the Quality Control (QC) module of RiboMiner. **a** 3-nt periodicity plots generated by RiboCode and RiboMiner, using the reads of 28 nt as an example. **b** Length distributions of all the RPF reads and the reads mapped to different genomic regions including exons, intergenic regions, and introns. **c** Numbers of reads mapped to different regions of the genome. **d** Reads mapped to different reading frames. The sample SRR5008135 in the dataset GSE89704 was used as an example here

Benchmarks for the quality of a ribosome profiling dataset include 3-nt periodicity, appropriate distribution of the footprint lengths (usually ~ 28–30 nt), and enrichment in coding regions. As for GSE89704 (SRR5008135 for example), RiboMiner exhibited strong 3-nt periodicity for the reads with a specific length or for all the reads combined and aligned by their P-site positions (Fig. 3a). The lengths of ribosome footprints are usually 28 ~ 30 nt [1]. However, the footprint length distribution of SRR5008135, generated by RiboMiner, showed that although the main peak was indeed around 28–30 nt, there is a small peak at 19 nt (Fig. 3b), which seems quite abnormal. This was further addressed by genome mapping of the RPF reads by RiboMiner, which showed that a small proportion of the reads were mapped to the intergenic regions and introns, suggesting some level of potential DNA contamination (Fig. 3b, c). After removal of these reads, the footprints strongly unified around 28–30 nt. Finally, frame analysis of the footprints showed that most reads were enriched in the first reading frame (Fig. 3d). Taken together, the results above indicate generally good quality of the data and identified the source of potential contamination, which could be easily eliminated by size selection of the raw reads.

### Metagene analysis

**Fig. 4:**
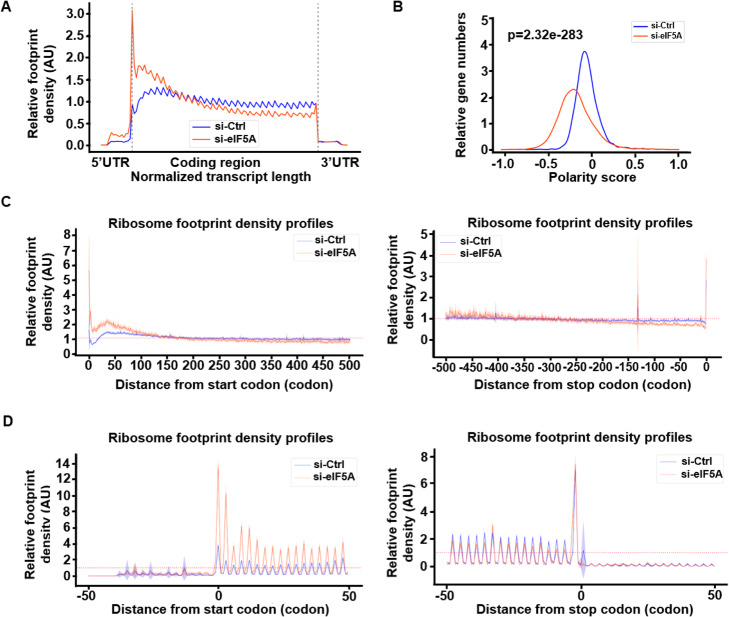
Results of Metagene Analysis (MA) by RiboMiner. **a** Overall read density distributions along all the transcripts merged. **b** Distributions of polarity scores. **c** Ribosome densities along the CDS regions. Left: read density after the start codon. Right: read density before the stop codon. **d** Ribosome densities along the UTR regions. Left: read density around the start codon. Right: read density around the stop codon. Shading area represents 95% confidence interval

RiboMiner was used for metagene analysis of the ribosome footprint distributions with a similar procedure as previously described [8]. As shown in Fig. 4a, RiboMiner reproduced the dramatically changed pattern of ribosome occupancy upon knock-down of eIF5A, suggesting strong stalling at the early stage of elongation in the first 100 ~ 150 codons (Fig. 4a, c). Distributions of the polarity scores, generated by RiboMiner, confirmed the significant shift of the ribosome footprints towards the 5′ ends (Fig. 4b). In addition to the metagene analysis along the whole transcript or the CDS region, RiboMiner could also be used for metagene analysis for the UTR regions (Fig. 4d). Finally, it is worth noting that the metagene analysis with RiboMiner can be done at the global scale or just for a subset of the genes.

### Mining of features related to translation regulation

Although the global metagene analysis as shown above indicates strong ribosome stalling upon eIF5A knock-down (Fig. 4a, c), it is possible that such pattern was mainly contributed by a subset of the genes. To specifically identify the genes with ribosome footprints enriched in the first 100 codons upon eIF5A knock-down, we compared the read densities on the first 100 codons for each transcript between the control and si-eIF5A samples. Two thousand nine hundred fifty-four genes showed up-regulated ribosome densities (“up-regulated genes”) with the ratio of si-eIF5A/si-Ctrl > = 1.5. This list of genes could then be used for mining of common features that are potentially related to the ribosome stalling during early elongation.

**Fig. 5:**
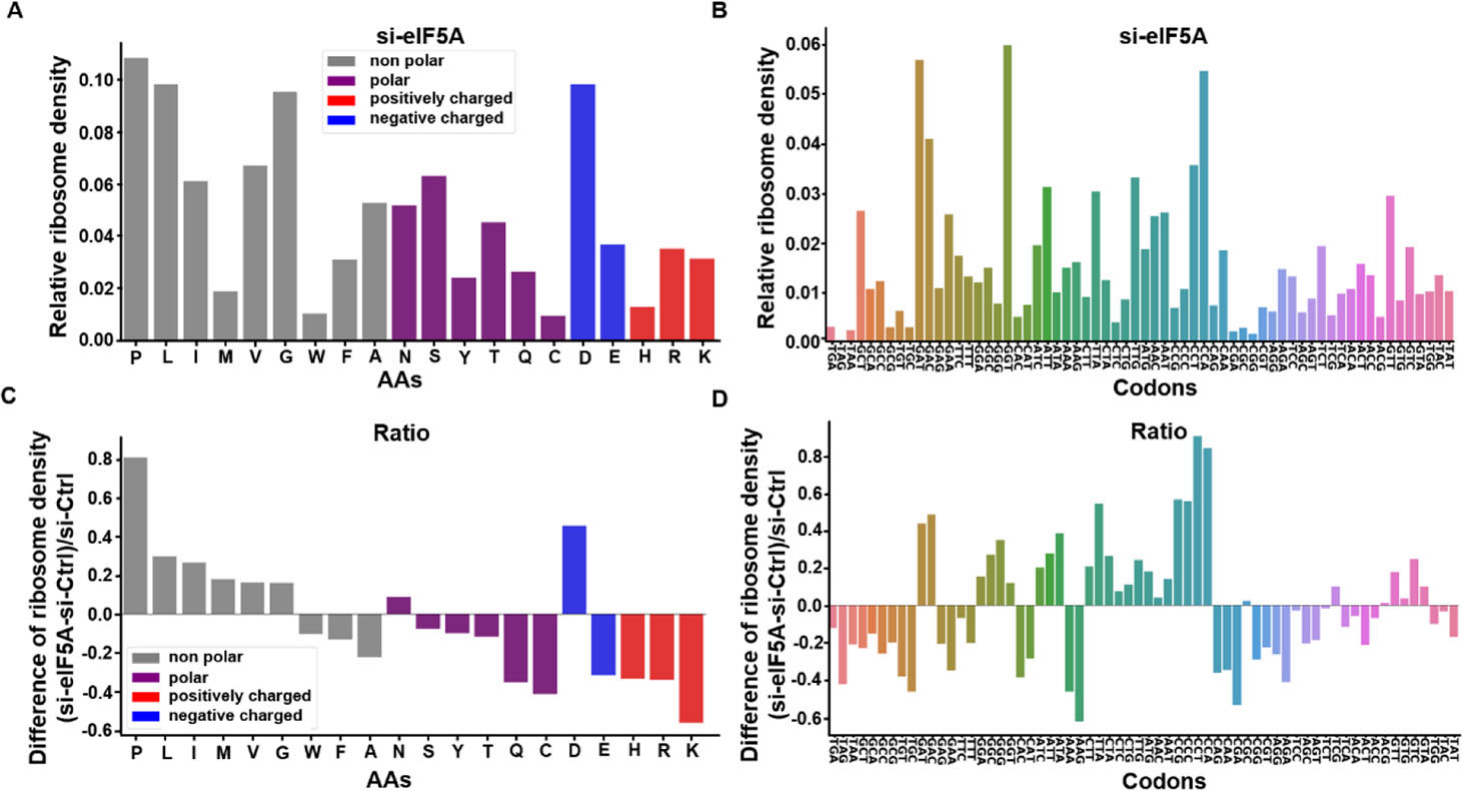
Comparison of the ribosome densities among different amino acids and codons. **a** Relative ribosome density on each amino acid for the sample of si-eIF5A. **b** Relative ribosome densities on different codons for the si-eIF5A sample. **c** The ratio (log2) of ribosome densities for each amino acid in the sample of si-eIF5A over si-Ctrl. **d** The ratio (log2) of ribosome densities for each codon in the sample of si-eIF5A over si-Ctrl. All the analyses above were based on the 2954 up-regulated transcripts. Ribosome densities were calculated with reads aligned by their P-sites

For example, RiboMiner can calculate ribosome density at each codon or amino acid (AA) for a set of genes (Fig. 5a, b) and further evaluate the change of ribosome density under different conditions (Fig. 5c, d). The results showed that read densities on proline (P) and aspartic acid (D) were largely increased after eIF5A-KD (si-eIF5A), suggesting that these amino acids may have contributed to the ribosome stalling observed above.

**Fig. 6:**
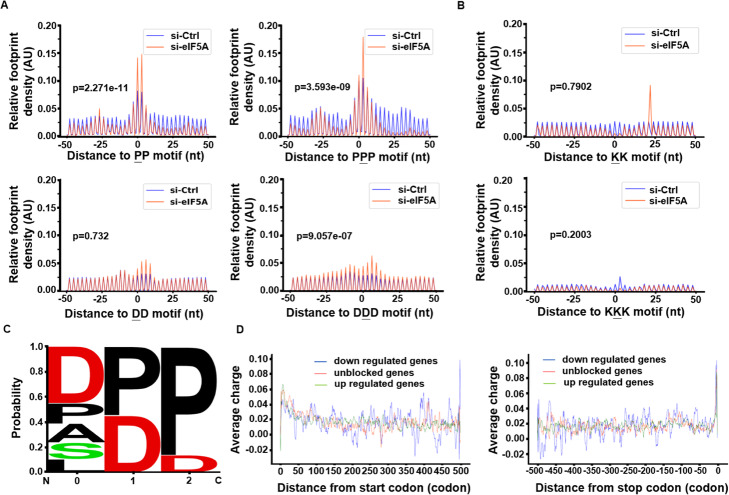
Ribosome densities among tri-AA motifs. **a** Relative ribosome densities on poly-proline and poly-Aspartic acid. **b** Relative ribosome densities on poly-Lysine. **c** The tri-AA motifs with enriched ribosome, reported by RiboMiner. 0,1,2 represents E, P, A site of a tri-AA motif, respectively. **d** Average charge of the amino acids encoded by each codon along the transcripts. All the analyses above were based on the 2954 up-regulated transcripts

Next, RiboMiner was also used for calculating the ribosome densities on tri-AA motifs. Results showed that the motifs of poly-proline (PPP) and poly-aspartic acid (DDD) were significantly enriched by ribosome upon eIF5A knock-down (Fig. 6a), whereas the ribosome density on poly-lysine was decreased, but with no statistical significance (Fig. 6b). These results are consistent to the original reports in [8] (Fig. 6c). Besides, although the charges of amino acids have been reported to be negatively correlated to the speed of translation elongation [43], we found no difference in the charge of amino acids among the different gene sets (Fig. 6d).

**Fig. 7:**
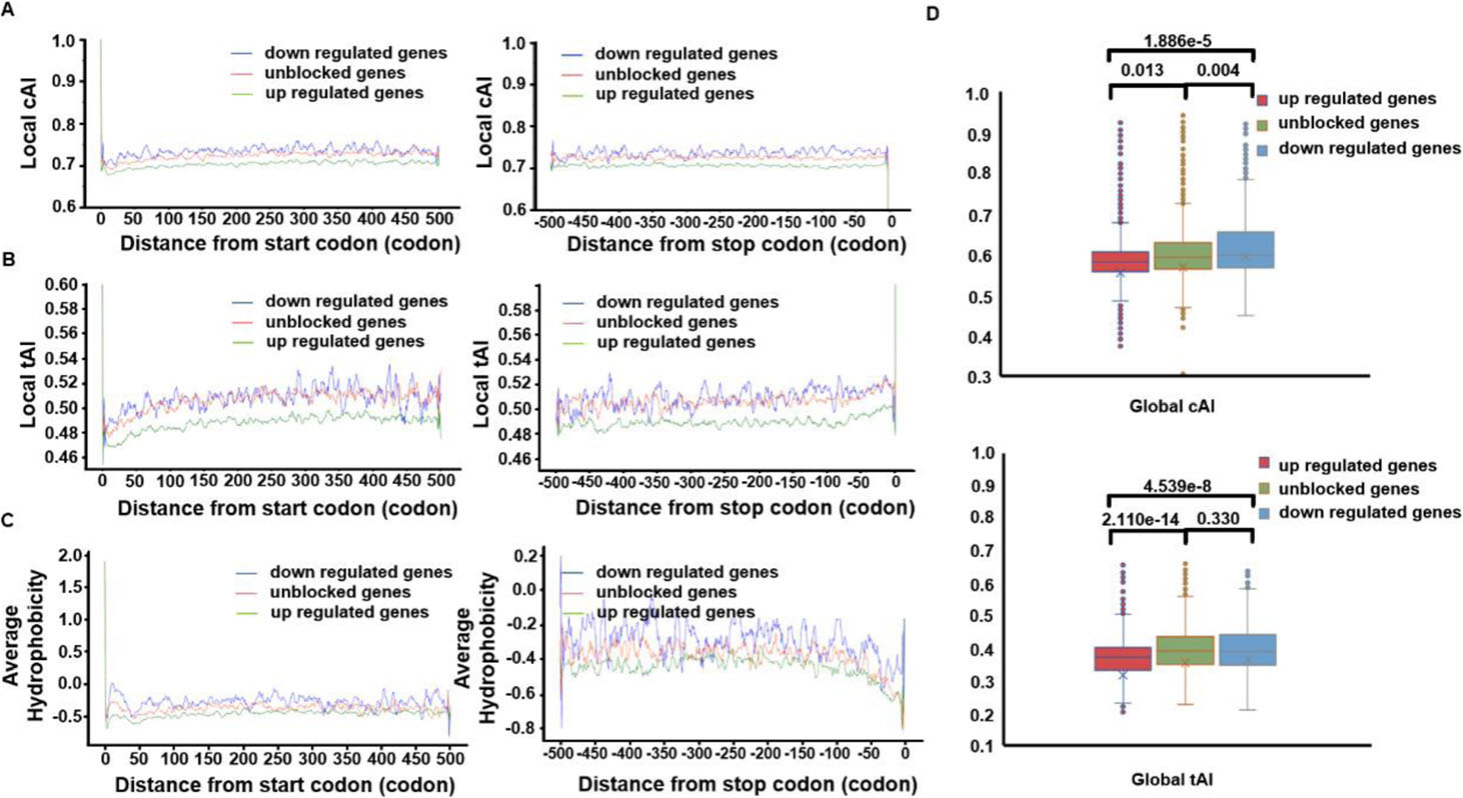
Results of Feature Analysis (FA) by RiboMiner. **a** Distributions of local cAI along different gene sets. **b** Distributions of local tAI along different gene sets. **c** Average hydrophobicity of each amino acid encoded along the transcripts of different gene sets. **d** Distributions of global cAI and global tAI for different gene sets. The *P*-values with T-tests are provided on the plots

Interestingly, analysis with RiboMiner revealed some more novel features that have not been reported in the original study. For example, we found that both the local and global cAI and tAI values of the genes with ribosome enrichment during early elongation (“up-regulated gene”) tend to be much smaller than those of the other transcripts used as control (“unblocked genes” and “down-regulated genes”) and so did the local cAI and tAI values (Fig. 7a, b, d). This suggests that the “up-regulated genes” have more sub-optimal codons, which then potentially led to slower moving of the ribosomes along the transcripts. In addition, the hydrophobicity of the amino acids encoded by these “up-regulated genes” are also much smaller, which we suspect may be related to ribosome stalling as well (Fig. 7c).

In summary, based on the results of RiboMiner, we reproduced the ribosome stalling event upon eIF5A knock-down as reported by the original study. Such stalling was mainly contributed by a subset of genes, and further mining of multiple features of these genes indicated that tri-AA motifs, codon usage, tRNA copy numbers, and amino acid hydrophobicity may be related to the translation dysregulations due to eIF5A knock-down. Many of these new insights have not been reported by the original study and therefore could worth further investigations.

### Enrichment analysis with selective ribosome profiling data

**Fig. 8:**
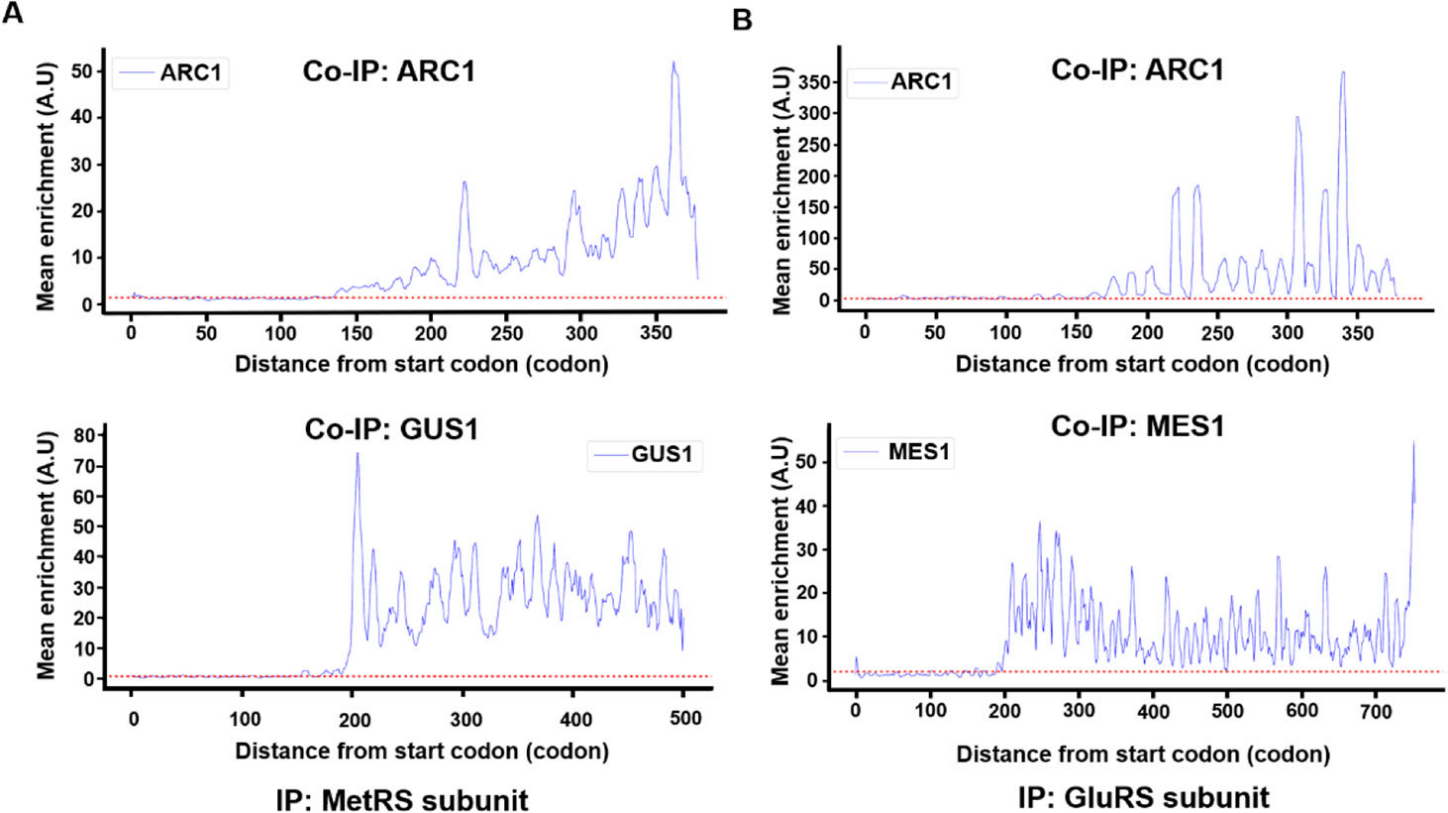
Results of Enrichment Analysis by RiboMiner. **a** Engagement of nascent ARC1 (top), GUS1 (bottom) by C-terminally tagged MetRS. **b** Engagement of nascent ARC1 (top), MES1 (bottom) by C-terminally tagged GluRS. The red dotted lines represent the two-fold threshold

We used RiboMiner to revisit the selective ribosome profiling data in the study of the assembly of a hetero-trimeric complex, the multi-aminoacyl-tRNA synthetase [47]. This complex is composed of three major subunits, i.e., the essential methionyl- and glutamyl-tRNA synthetases MetRS and GluRS (encoded by *MES1* and *GUS1*, respectively) and the Arc1p cofactor (encoded by *ARC1*) regulating the catalytic activities and subcellular distributions of the complex [47]. RiboMiner regenerated the distributions of ribosome footprints that are enriched in the selective ribosome profiling (Fig. 8). This confirms the main conclusion of the original study, i.e., proteins MetRS and GluRS could co-translate with each other, and both participate in the translation of Arc1p starting at a specific position [47].

### Running time of different modules of RiboMiner

Last, the running time of each module of RiboMiner with the data of 4 different model organisms are provided in Table 1. We did not consider the pre-filtering and data preparation steps. In general, the time mainly depends on the total reads, i.e., the sequencing depth. All jobs were run on a Linux server with Intel R Xeon R CPUs at 2.40GHz, 64 G memory. Note that the table reports the total running time of all the functions in each module. The scripts and results for evaluation of the running time are available at https://github.com/xryanglab/RiboMiner/tree/master/RuningTimeTest.

**Table 1:** Computation time of each module in RiboMiner for different organisms

Modules	Yeast	Fly	Mouse	Human
Data source	GSE89704	GSE79626	GSE32060	GSE59821
Test samples	SRR5008135/ SRR5008137	SRR3297802/ SRR3297804	SRR649755/ SRR649757	SRR2873530/ SRR2873532
Unique mapped reads	16,318,577/ 18,441,750	7,627,569/ 12,099,927	16,457,639/ 7,282,998	11,557,500/ 12,896,290
QC	32.2 min	16.3 min	35.1 min	29.2 min
MA	24.6 min	20.0 min	14.2 min	22.7 min
FA	21.6 min	17.7 min	14.6 min	20.0 min
EA	2.4 min	2.4 min	2.4 min	3.4 min
Total	80.8 min	56.4 min	66.3 min	75.3 min

## Conclusions

RiboMiner is a python toolset for deep mining of multi-dimensional features of the translatomes with ribosome profiling data. A multitude of functions incorporated in RiboMiner are useful for quality control of ribosome profiling data, metagene analysis for detection of translation dysregulations such as ribosome stalling, mining of various features related to the translation dysregulations, and exploration of selective ribosome profiling for fine maps of translation regulation such as co-translation. Applications of RiboMiner on two published datasets did not only reproduce the main results reported before, but also generated novel insights into the translation regulation processes. RiboMiner provides quantitative data for visualization as well as statistical analyses. In summary, here we present RiboMiner as a complementary toolset to the current methods, to facilitate the comprehensive and thorough dissections of the translation landscapes as well as the translation regulations with the technique of ribosome profiling.

## Availability and requirements

**Project name:** RiboMiner

**Project home page:**https://github.com/xryanglab/RiboMiner

**Operating system(s):** Platform independent

**Programming language:** Python

**Other requirements:** Python (version > = 3.6)

**License:** GNU GPL

**Any restrictions to use by non-academics:** Licence needed

## Supplementary information

**Additional file 1.**

## Data Availability

RiboMiner is freely available at https://github.com/xryanglab/RiboMiner and https://pypi.org/project/RiboMiner. Detailed instructions and the information of all the sequencing data used in the current manuscript are available at https://github.com/xryanglab/RiboMiner/blob/master/Implementation.md. We also offer a Docker image for RiboMiner at https://hub.docker.com/r/yanglab/ribocode_ribominer. The RiboMiner pipeline, including the testing data used in the present study, is also available as a Gene Container Service (GCS) on the Huawei Cloud.

## Abbreviations

TE: Translation efficiency
ORFs: Open reading frames
TIS: Translation initiation site
QC: Quality control
MA: Metagene analysis
FA: Feature analysis
EA: Enrichment analysis
AA: Amino acid
RPF: Ribosome profiling footprint
UTR: Un-translated region
CDS: Coding sequence
tAI: tRNA adaptation index
cAI: Codon adaptation index
SeRP: Selective ribosome profiling
