# Supplementary material for: RiboMiner: a toolset for mining multi-dimensional features of the translatome with ribosome profiling data
Source: BMC Bioinformatics. 2020 Aug 1;21:340. doi: 10.1186/s12859-020-03670-8 (PMC7430821; doi:10.1186/s12859-020-03670-8)
Supplement: Supplementary file 1 — Additional file 1. [file 12859_2020_3670_MOESM1_ESM.docx]

**RiboMiner: a toolset for** **mining multi-dimensional features of the translatome with ribosome profiling data**

Fajin Li^1,2,3^, Xudong Xing^1,2,3^, Zhengtao Xiao^1,2^, Gang Xu^1^, and Xuerui Yang^1,2^

^1^ MOE Key Laboratory of Bioinformatics, School of Life Sciences, Tsinghua University, Beijing 100084, China

^2^ Center for Synthetic & Systems Biology, Tsinghua University, Beijing 100084, China

^3^ Joint Graduate Program of Peking-Tsinghua-National Institute of Biological Science, Tsinghua University, Beijing 100084, China.

**Corresponding author:** Xuerui Yang, Medical Science Building D231, School of Life Sciences, Tsinghua University, Beijing 100084, China. Tel: 86-10-62783943. Email: [yangxuerui@tsinghua.edu.cn](mailto:yangxuerui@tsinghua.edu.cn)

**Contents**

[**Introduction 3**](#_Toc21875040)

[**Data preparation (DP) 3**](#_Toc21875041)

[**Datasets download 3**](#_Toc21875042)

[**Prepare annotation file 3**](#_Toc21875043)

[**Preprocessing 4**](#_Toc21875044)

[**Quality control 4**](#_Toc21875045)

[**Adapter trimming 4**](#_Toc21875046)

[**Quality filtering 5**](#_Toc21875047)

[**Remove rRNA contamination 5**](#_Toc21875048)

[**Quality control 5**](#_Toc21875049)

[**Reads mapping 5**](#_Toc21875050)

[**RiboMiner 6**](#_Toc21875051)

[**Quality Control (QC) 6**](#_Toc21875052)

[**Metagene Analysis (MA) 8**](#_Toc21875053)

[**Feature Analysis (FA) 11**](#_Toc21875054)

[**Enrichment Analysis (EA) 19**](#_Toc21875055)

[**Methods 21**](#_Toc21875056)

[**Pre-processing of the ribosome profiling data 21**](#_Toc21875057)

[**Quality control of the ribosome profiling data 22**](#_Toc21875058)

[**Metagene analyses of the ribosome profiling data 22**](#_Toc21875059)

[**Polarity calculation 23**](#_Toc21875060)

[**Read density at each codon and amino acid 23**](#_Toc21875061)

[**Hydrophobicity index 23**](#_Toc21875062)

[**Charges of amino acid 24**](#_Toc21875063)

[**The local tAI and global tAI 24**](#_Toc21875064)

[**The local cAI and the global cAI 24**](#_Toc21875065)

[**Enrichment analysis 25**](#_Toc21875066)

[**Reference 25**](#_Toc21875067)

# Introduction

The [RiboMiner](https://github.com/xryanglab/RiboMiner) is a python toolset for mining multi-dimensional features of the translatome with ribosome profiling data. This package has four function parts:

- **Quality Control (QC)**: Quality control for ribosome profiling data, containing periodicity checking, reads distribution among different reading frames, length distribution of ribosome footprints and DNA contaminations.
- **Metagene Analysis (MA**): Metagene analysis among different samples to find possible ribosome stalling events.
- **Feature Analysis (FA)**: Feature analysis among different gene sets identified in MA step to explain the possible ribosome stalling.
- **Enrichment Analysis (EA)**: Enrichment analysis to find possible co-translation events.

In this file, we will show how to use [RiboMiner](https://github.com/xryanglab/RiboMiner) based on some published datasets. Please refer to CodeOcean (<https://codeocean.com/capsule/3780896/tree/v1>) for more details. The RiboMiner pipeline, including the testing data used in the present study, is also available as a Gene Container Service (GCS) on the Huawei Cloud.

# Data preparation (DP)

## Datasets download

we will use [GSE89704](https://www.ncbi.nlm.nih.gov/geo/query/acc.cgi?acc=GSE89704) and [GSE116570](https://www.ncbi.nlm.nih.gov/geo/query/acc.cgi?acc=GSE116570) datasets for following analysis. Genome sequence file and annotation file were downloaded from [ensemble](http://www.ensembl.org/Saccharomyces_cerevisiae/Info/Index).

*## for GSE89704
for i in SRR50081{34..37};do fastq-dump $i;done
## for GSE116570
for i in SRR74712{49..56};do fastq-sump $i;done*

## Prepare annotation file

- **Prepare sequences and annotaiton files on transcriptome level.**

*prepare_transcripts -g Saccharomyces.gtf -f Saccharomyces_genome.fa -o RiboCode_annot*

Because there is almost no UTR regions for Saccharomyces cerevisiae, for better observation I extended 50 nt away from start codon and stop codon, which means coordinates in gtf file are increased about 50 nt for both start and end position. *prepare_transcripts* is the function of RiboCode our lab developed before.

- **Prepare the longest transcript annotaion files.**

*## command*
*OutputTranscriptInfo -c RiboCode_annot/transcripts_cds.txt -g Saccharomyces.gtf -f RiboCode_annot/transcripts_sequence.fa -o longest.transcripts.info.txt -O all.transcripts.info.txt*
*## output*
*Starting outputing longest trans...*
*6692 transcripts will be used in the follow analysis.*
*Finishing!*
*Starting outputing all trans...*

- **Prepare the sequence file for the longest transcripts**

*GetProteinCodingSequence -i RiboCode_annot/transcripts_sequence.fa -c longest.transcripts.info.txt -o <output_prefix> --mode whole --table 1
##
6692 transcripts will be used in the follow analysis.
Notes: There are 0 transcripts whose cds sequence cannot be divided by 3!
Finish the step of extracting sequences!*

# Preprocessing

The Preprocessing step contains some basic steps, such as quality control, adapters trimming, quality filtering, removing rRNA contamination, reads mapping, et al. Here we used part of samples from [GSE89704](https://www.ncbi.nlm.nih.gov/geo/query/acc.cgi?acc=GSE89704) as an example.

## Quality control

*fastqc SRR5008135.fastq -o <outputDir>*

## Adapter trimming

*cutadapt -m 15 -M 35 --match-read-wildcards -a CTGTAGGCACCATCAAT -o SRR5008135.trimmed.fastq SRR5008135.fastq > SRR5008135_trimmed.log*

## Quality filtering

*fastq_quality_filter -Q33 -v -q 25 -p 75 -i SRR5008135.trimmed.fastq -o SRR5008135.trimmed.Qfilter.fastq > SRR5008135.Qfilter.log*

## Remove rRNA contamination

*#!/bin/bash
bowtie_noncoding_index=/Share2/home/lifj/Reference/yeast/saccharomyces/Bowtie_noncoding_index/saccharomyces_noncoding
bowtie -n 0 -y -a --norc --best --strata -S -p 4 -l 15 --un=noncontam_SRR5008135.fastq $bowtie_noncoding_index -q SRR5008135.trimmed.Qfilter.fastq SRR5008135.alin*

## Quality control

This step is a **Quality control** step based on *fastq* files generated on the **Remove rRNA contamination** step, after which the reads filtered will be used for mapping.

## Reads mapping

*## use one sample for example
#!/bin/bash
#BSUB -J STAR.sh
#BSUB -n 8
STAR_genome_index=/Share2/home/lifj/Reference/yeast/saccharomyces/STAR_genome_extended_index
workdir=/Share2/home/lifj/Projects/03.RiboMiner/2017_eIF5A/07.STAR
fastqFile=/Share2/home/lifj/Projects/03.RiboMiner/2017_eIF5A/05.contam
i=SRR5008134
mkdir -p $workdir/${i}_STAR
## mapping
STAR --runThreadN 8 --outFilterType Normal --outWigType wiggle --outWigStrand Stranded --outWigNorm RPM --alignEndsType EndToEnd --outFilterMismatchNmax 1 --outFilterMultimapNmax 1 --genomeDir $STAR_genome_index --readFilesIn $fastqFile/noncontam_$i.fastq --outFileNamePrefix $workdir/${i}_STAR/$i. --outSAMtype BAM SortedByCoordinate --quantMode TranscriptomeSAM GeneCounts --outSAMattributes All
## sort
samtools sort -T $workdir/${i}_STAR/$i.Aligned.toTranscriptome.out.sorted -o $workdir/${i}_STAR/$i.Aligned.toTranscriptome.out.sorted.bam $workdir/${i}_STAR/$i.Aligned.toTranscriptome.out.bam
## index
samtools index $workdir/${i}_STAR/$i.Aligned.toTranscriptome.out.sorted.bam ## mapped to transcriptome
samtools index $workdir/${i}_STAR/$i.Aligned.sortedByCoord.out.bam ## mapped to genome*

# RiboMiner

## Quality Control (QC)

- **Checking 3-nt periodicity**

*## use RiboCode:metaplots (recommeded)
#!/bin/bash
#BSUB -q TEST-A
workdir=/Share2/home/lifj/Projects/03.RiboMiner/2017_eIF5A/08.periodicity
BamDir=/Share2/home/lifj/Projects/03.RiboMiner/2017_eIF5A/07.STAR
RiboCode_annot=/Share2/home/lifj/Reference/yeast/saccharomyces/RiboCode/RiboCode_annotate_extend
for i in SRR5008135 SRR5008136 SRR5008137 SRR5008134;
do
 metaplots -a $RiboCode_annot -r $BamDir/${i}_STAR/$i.Aligned.toTranscriptome.out.bam -o $workdir/$i
done
## use RiboMiner
#!/bin/bash
#BSUB -J run_Periodicity.sh
#BSUB -q TEST-A
workdir=/Share2/home/lifj/Projects/03.RiboMiner/2017_eIF5A/08.periodicity
bamFiles=/Share2/home/lifj/Projects/03.RiboMiner/2017_eIF5A/07.STAR
Ref=/Share2/home/lifj/Reference/yeast/saccharomyces
for i in SRR5008135 SRR5008136 SRR5008137 SRR5008134;do
 Periodicity -i $bamFiles/${i}_STAR/$i.Aligned.toTranscriptome.out.sorted.bam -a $Ref/RiboCode/RiboCode_annotate_extend -o $workdir/$i -c $Ref/longest.transcripts.info.txt -L 25 -R 35
done*

*metaplots* from [RiboCode](https://github.com/xryanglab/RiboCode) is used for 3-nt periodicity checking, and it will output a *pdf* file for plotting of distribution of reads with different length. However, it can not output the density of reads with different length which could not meet some specific analysis requirements. Therefore, we developed *Periodicity* to do the same thing as *metaplots* did but without P-site identification [(Figure 1A)](http://static.zybuluo.com/sherking/4wuu4omw1r3edhnhxoalot9q/QC2.png). And it could generate files used for periodicity plot as well as periodicity plot itself. In addition, *Periodicity* could also generate distribution plot of all reads rather than reads with a specific length [(Figure 1A)](http://static.zybuluo.com/sherking/4wuu4omw1r3edhnhxoalot9q/QC2.png).

Now that we have used *metaplots* checked the 3-nt periodicity of given sample, we should use the results to construct the **attributes.txt** file which will be used for following analysis.

*## attributes.txt
bamFiles readLengths Offsets bamLegends
./SRR5008134.bam 27,28,29,30 11,12,13,14 si-Ctrl-1
./SRR5008135.bam 27,28,29,30 11,12,13,14 si-Ctrl-2
./SRR5008136.bam 27,28,29 11,12,13 si-eIF5A-1
./SRR5008137.bam 27,28,29 11,12,13 si-eIF5A-2*

- **Reads distribution among different reading frames.**

A ribosome profiling data (RPF data) with a good 3-nt periodicity tend to enrich ribosomes at a specific reading frame. RiboDensityOfDiffFrames was developed to do such jobs.

*RiboDensityOfDiffFrames -f attributes.txt -c longest.transcripts.info.txt -o <output_prefix> --plot yes*

The output of this step would generate two files. One is the reads distribution of different reading frames [(Figure 1D)](http://static.zybuluo.com/sherking/4wuu4omw1r3edhnhxoalot9q/QC2.png) and the other is the read density file used for generating this kind of plot.

- **Length distribution**

The length distribution of normal ribosome profiling data is around 28nt~30nt， any abnormal length distribution maybe represent problems from library construction or sequencing steps. So this is also an important criterian for data with a good quality.

*LengthDistribution -i SRR5008135.trimmed.Qfilter.fastq -o SRR5008135*

The length distribution of SRR5008135 sample was shown on [(Figure 1B)](http://static.zybuluo.com/sherking/4wuu4omw1r3edhnhxoalot9q/QC2.png) with a *Total* tag. As what we can see, most reads are about 28~30 nt, but there seems to be a small peak on 18~19 nt which is quite strange. To check what those reads are, we did such statistics for reads mapped to different regions of genome.

- **Checking DNA contamination.**

*StatisticReadsOnDNAsContam -i SRR5008135.Aligned.sortedByCoord.out.bam -g Saccharomyces.gtf -o <output_prefix>*

The results showed that reads mapped to transcriptome are around 28~30 nt [(Figure 1B)](http://static.zybuluo.com/sherking/4wuu4omw1r3edhnhxoalot9q/QC2.png), and there are also some reads with this read length mapped to Intron and DNA intergenic region. Moreover, it is expected that those reads with length covered on 18~19 nt are DNA contaminations, which maybe suggest there are some problems on the step of library construction. In addition to the distribution plot of read length, this step would also generate a file containing the number of reads uniquely mapped to different regions [(Figure 1C)](http://static.zybuluo.com/sherking/4wuu4omw1r3edhnhxoalot9q/QC2.png).

## Metagene Analysis (MA)

- **Metagene analysis along the whole transcript region.**

*MetageneAnalysisForTheWholeRegions -f attributes.txt -c longest.transcripts.info.txt -o <output_prefix> -b 15,90,15 -l 100 -n 10 -m 1 -e 5*

This step is used for checking whether there is a global ribosome enrichment on transcriptome under a specific condition. As for [**GSE89704**](https://www.ncbi.nlm.nih.gov/geo/query/acc.cgi?acc=GSE89704) dataset, just as what has been described, there are more ribosomes enriched on the early elongation stage after the expression of eIF5A was interfered [(Figure 2A)](http://static.zybuluo.com/sherking/v59lhdcvwk57fya119a4r3rq/MA.png). This step would generate a metagene results with a scaled transcript length. Use *PlotMetageneAnalysisForTheWholeRegions* could generate the metagene plot.

*PlotMetageneAnalysisForTheWholeRegions -i <output_prefix_scaled_density_dataframe.txt> -o <output_prefix> -g si-Ctrl,si-eIF5A -r si-Ctrl-1,si-Ctrl-2__si-eIF5A-1,si-eIF5A-2 -b 15,90,15 --mode all --xlabel-loc -0.4*

Note: the *-b* parameter must be consistent with that in *MetageneAnalysisForTheWholeRegions*. But this is a general observation of reads distribution of different regions along transcripts. If one want to locate the specific position of ribosome stalling, *MetageneAnalysis* could be helpful.


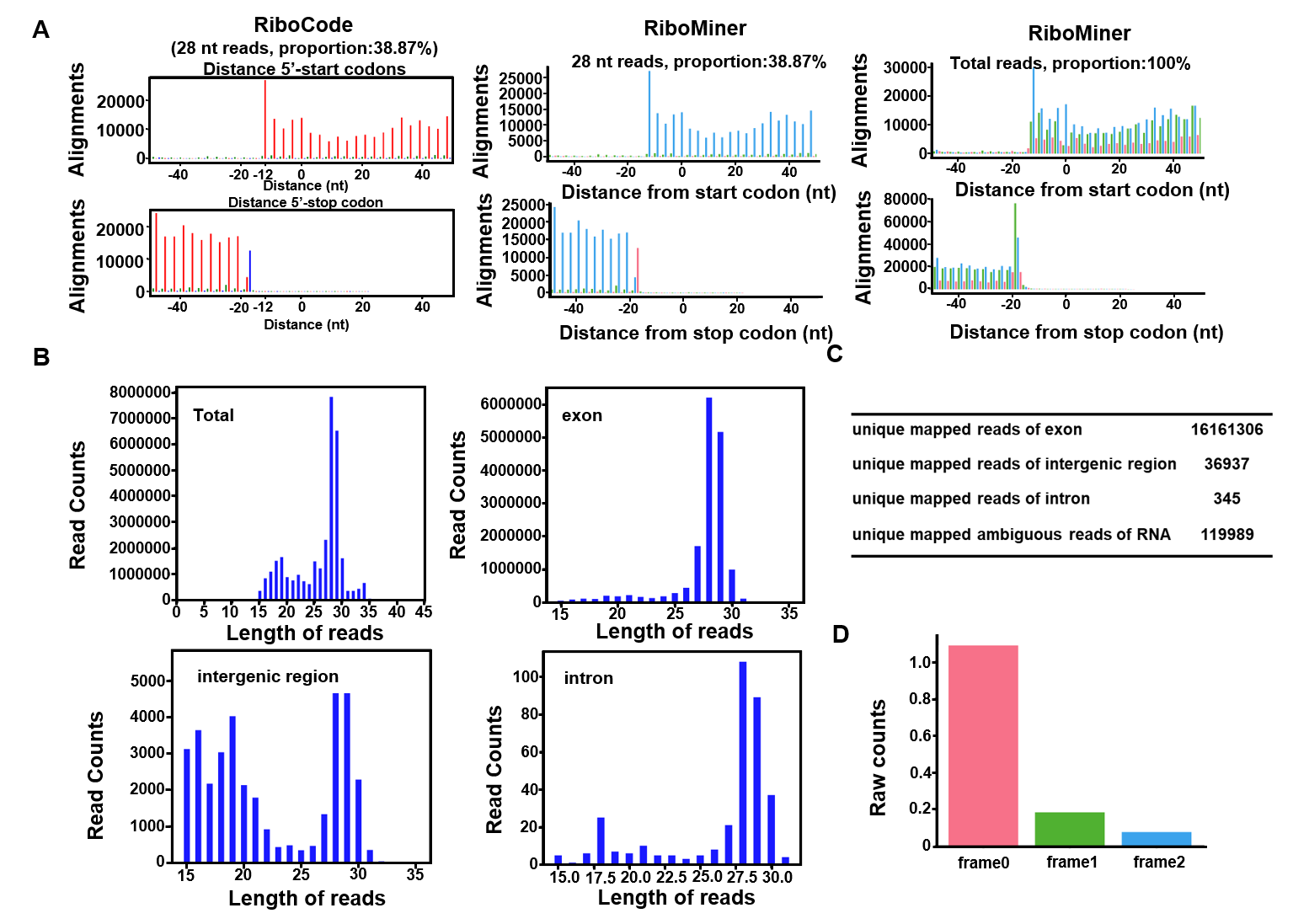


**Figure 1: Results of the Quality Control (QC) module of RiboMiner**. **(A)** 3-nt periodicity plots generated by RiboCode and RiboMiner, using the reads of 28 nt for example. **(B)** Length distributions of all the RPF reads and the reads mapped to different genomic regions including exons, intergenic regions, and introns. **(C)** Number of reads mapped to different regions of the genome. **(D)** Reads mapped to different reading frames. The sample SRR5008135 in the dataset GSE89704 was used as an example here.

- **Metagene analysis on CDS regions.**

*## metagene analysis
MetageneAnalysis -f attributes.txt -c longest.transcripts.info.txt -o <output_prefix> -U codon -M RPKM -u 0 -d 500 -l 100 -n 10 -m 1 -e 5 --norm yes -y 100 --CI 0.95 --type CDS
## plot
PlotMetageneAnalysis -i <output_prefix_CDS_normed_dataframe.txt> -o <output_prefix> -u 0 -d 500 -g si-Ctrl,si-eIF5A -r si-Ctrl-1,si-Ctrl-2__si-eIF5A-1,si-eIF5A-2 -U codon --CI 0.95*

This would generate plots that could help us locate the range of the general ribosome stalling [(Figure 2C)](http://static.zybuluo.com/sherking/v59lhdcvwk57fya119a4r3rq/MA.png). As you can see that, the range of ribosome stalling happened to yeast cell under the condition of eIF5A interfered was around 0-150 codons which was a little different from the results described from the [original paper](https://www.ncbi.nlm.nih.gov/pubmed/28392174).

- **Metagene analysis on UTR regions.**

*## metagene analysis for UTR
MetageneAnalysis -f attributes.txt -c longest.transcripts.info.txt -o <output_prefix> -U nt -M RPKM -u 50 -d 50 -l 50 -n 10 -m 1 -e 5 --norm yes -y 100 --CI 0.95 --type UTR
## plot
PlotMetageneAnalysis -i <output_prefix_UTR_dataframe.txt> -o <output_prefix> -u 50 -d 50 -g si-Ctrl,si-eIF5A -r si-Ctrl-1,si-Ctrl-2__si-eIF5A-1,si-eIF5A-2 -U nt --CI 0.95*

We can also explore the read distribution on UTR regions along transcripts with *–type* reset to *UTR*. And the results were showed on [(Figure 2D)](http://static.zybuluo.com/sherking/v59lhdcvwk57fya119a4r3rq/MA.png).

- **Polarity calculation.**

*## polarity calculation
PolarityCalculation -f attributes.txt -c longest.transcripts.info.txt -o <output_prefix> -n 64
## plot
PlotPolarity -i <output_prefix_polarity_dataframe.txt> -o <output_prefix> -g si-Ctrl,si-eIF5A -r si-Ctrl-1,si-Ctrl-2__si-eIF5A-1,si-eIF5A-2 -y 5*

The way of polarity calculation is just like the [original paper](https://www.ncbi.nlm.nih.gov/pubmed/28392174). As you can see that, after the expression of eIF5A was interfered, reads seemed to be enriched more at the 5’ end of transcripts [(Figure 2B)](http://static.zybuluo.com/sherking/v59lhdcvwk57fya119a4r3rq/MA.png).


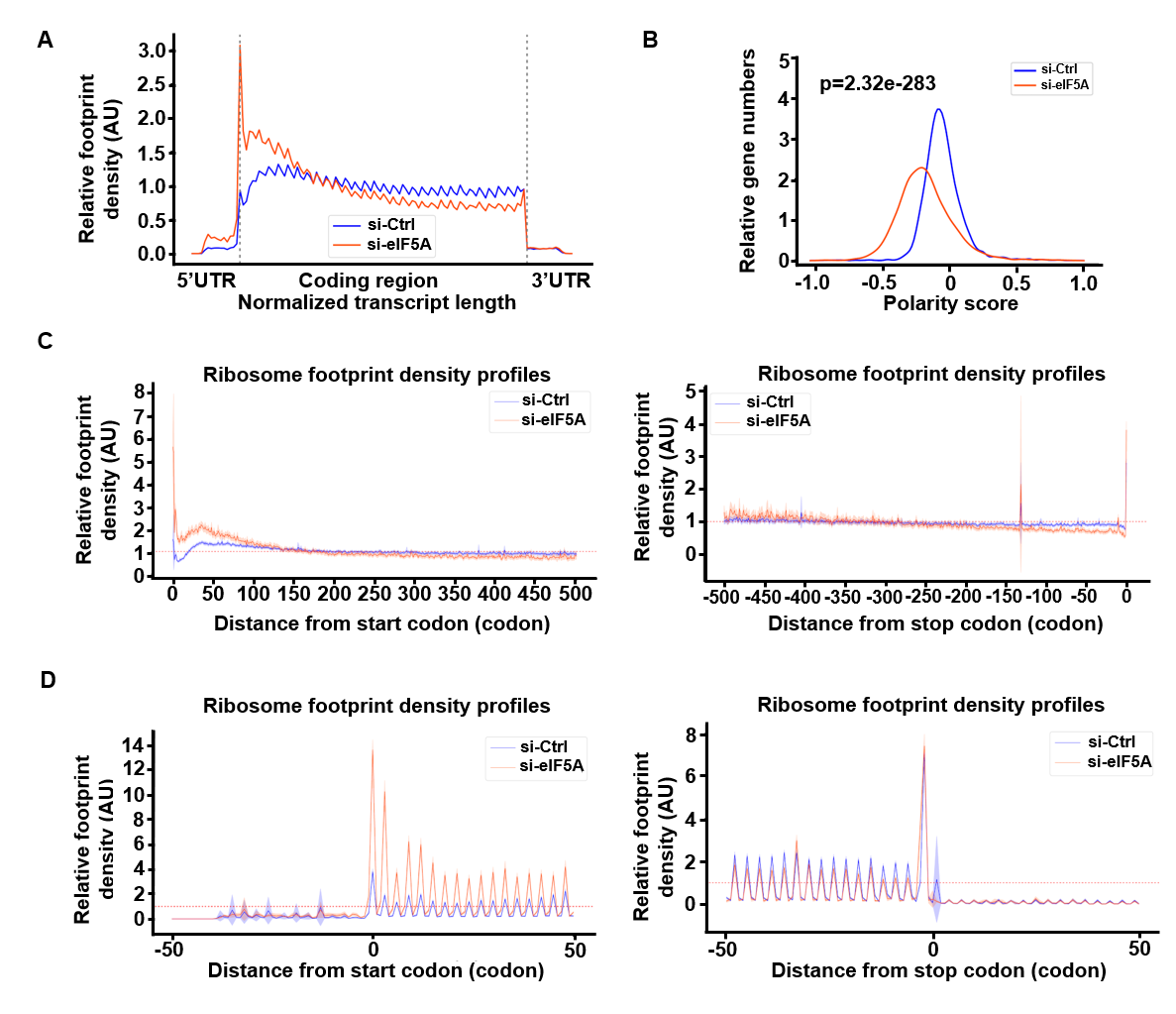


**Figure 2: Results of Metagene Analysis (MA) by RiboMiner.** (**A)**. A general read density distribution along transcripts. (**B)**. Distribution of polarity scores. (**C)**. Ribosome density profiles along CDS regions. left: read density after start codon. right: read density before stop codon. (**D)**. Ribosome density profiles along UTR regions. left: read density around start codon. right: read density around stop codon. Shading area represents 95% confidence interval.

## Feature Analysis (FA)

- **Pick out transcripts enriched ribosomes on specific region.**

*RiboDensityForSpecificRegion -f attributes.txt -c longest.transcripts.info.txt -o <output_prefix> -U codon -M RPKM -L 1 -R 100*

Base on the results of metagene analysis, there maybe exist general ribosome stalling for all transcripts [(Figure 2A, 2C, 2D)](http://static.zybuluo.com/sherking/v59lhdcvwk57fya119a4r3rq/MA.png). However, there maybe only exist a subset of transcripts enriched ribosomes on the 5’ end of CDSs, because the effect of down regulated or un-affected transcripts was shielded by the up regulated transcripts. Therefore, we use *RiboDensityForSpecificRegion* to calculate read density at the first 100 codons based on which we selected 2954 up regulated transcripts (enriched more ribosomes), 1598 unbloced transcripts (enriched a little or no ribosomes), and 433 down regulated transcripts (ribosomes decreased).

- **Ribosome density at each kind of AA or codon.**

In order to observe whether there is any difference between different codons or amino acids (AAs), we calculate ribosome density at each kind of codon or AA like this:

*RiboDensityAtEachKindAAOrCodon -f <attributes.txt> -c <longest.transcripts.info.txt> -o <output_prefix> -M RPKM -S <select_trans.txt> -l 100 -n 10 --table 1 -F <longest_cds_sequence.fa>*

The output of this step is a file containing ribosome density at each kind of codon or amino acid like this:

*AA codon ctrl-1 ctrl-2 treat-1 treat-2
K AAA 0.026594288206047208 0.029364263341463245 0.015578462857160988 0.014649362490996175
N AAC 0.023361565164523757 0.025593616862523674 0.025286390202746506 0.025597799715113747*

We can also calculate the ribosome density of each kind of AA or codon at a specific region, for example codon 1-100 by add -u and -d parameters.

*RiboDensityAtEachKindAAOrCodon -f <attributes.txt> -c <longest.transcripts.info.txt> -o <output_prefix> -M RPKM -S <select_trans.txt> -l 100 -n 10 --table 1 -F <longest_cds_sequence.fa> -u 1 -d 100*

- **Ribosome density on amino acids with positive or negative charge**

*PlotRiboDensityAtEachKindAAOrCodon -i <output_prefix_all_codon_density.txt> -o <output_prefix> -g si-Ctrl,si-eIF5A -r si-Ctrl-1,si-Ctrl-2__si-eIF5A-1,si-eIF5A-2 --level AA*

Based on the results of *RiboDensityAtEachKindAAOrCodon* we could get ribosome density at each kind of AA [(Figure 3A)](http://static.zybuluo.com/sherking/rl0wsq9zbrgnjg5f8alz00p5/AADensity.png) or codon [(Figure 3B)](http://static.zybuluo.com/sherking/rl0wsq9zbrgnjg5f8alz00p5/AADensity.png) for each sample. And we could also get the ribosome density change ratio between treat groups and control groups for different AA [(Figure 3C)](http://static.zybuluo.com/sherking/rl0wsq9zbrgnjg5f8alz00p5/AADensity.png) and codon [(Figure 3D)](http://static.zybuluo.com/sherking/rl0wsq9zbrgnjg5f8alz00p5/AADensity.png). As what we can see on [(Figure 3C)](http://static.zybuluo.com/sherking/rl0wsq9zbrgnjg5f8alz00p5/AADensity.png), ribosome density on most non polar amino acid are increased, especially the proline, the ribosome density on which increased almost 80% after eIF5A was interfered. And ribosome density on most polar amino acid and amino acid with positive charge were decreased.


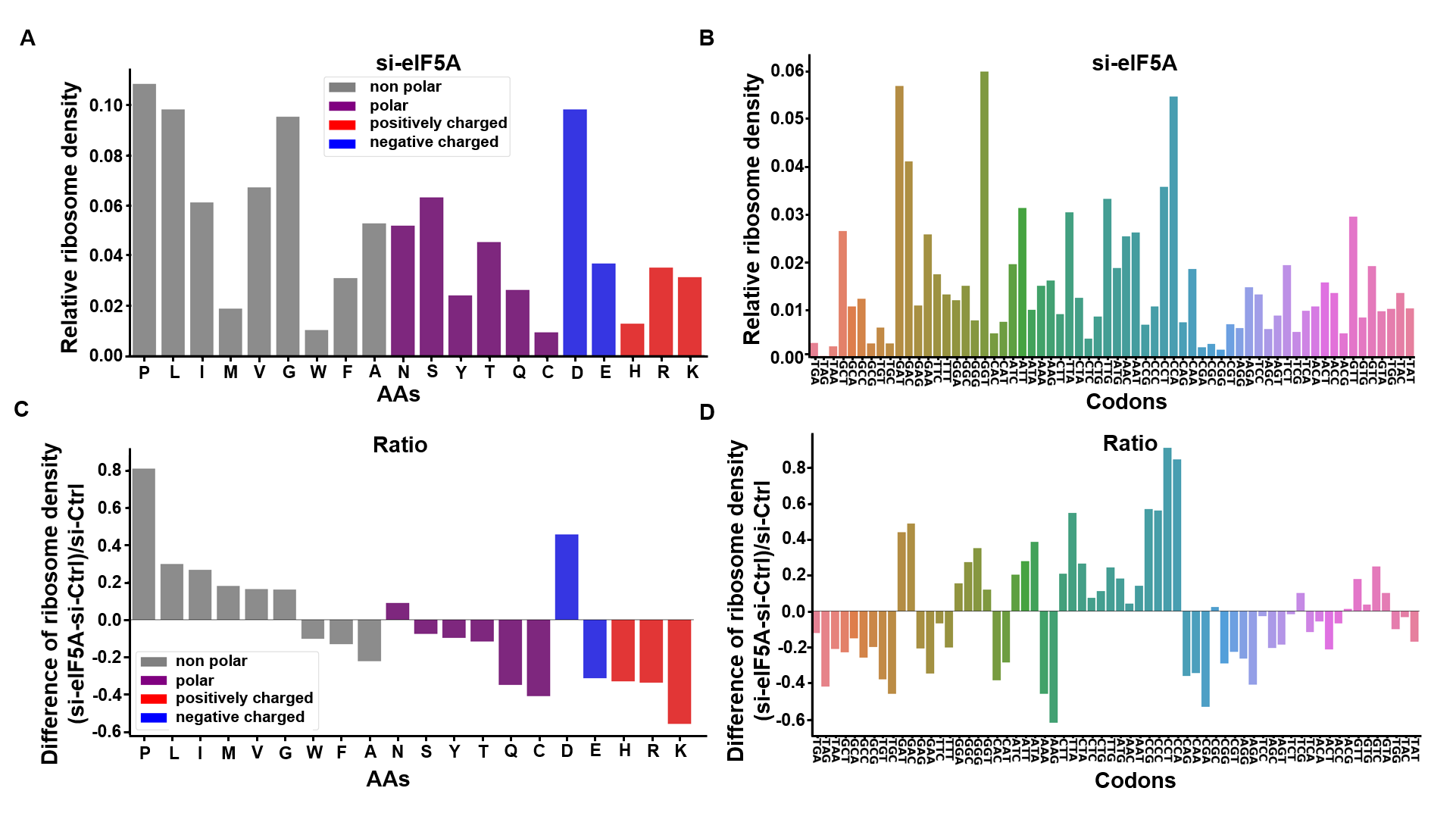


**Figure 3: Comparison of ribosome density among different amino acids and codons**. (**A).** Relative ribosome density on different amino acids for si-eIF5A sample. (**B).** Relative ribosome density on different codons for si-eIF5A sample. (**C)**. The change ratio of ribosome density between si-eIF5A sample and si-Ctrl sample for each amino acid. (**D).** The change ratio of ribosome density between si-eIF5A sample and si-Ctrl sample for each codon. **A**ll these analyses above are based on the 2954 up-regulated transcripts. Ribosome density are calculated by reads aligned with P-site.

- **Ribosome density around the triplete amino acid (tri-AA) motifs.**
- **As for a specific tri-AA motif, such as poly-proline (PPP)**

*## ribosome density at each tri-AA motif
RiboDensityAroundTripleteAAMotifs -f <attributes.txt> -c <longest.transcripts.info.txt> -o PPP -M RPKM -S <select_trans.txt> -l 100 -n 10 --table 1 -F <longest_cds_sequence.fa> --type2 PPP --type1 PP
## plot
PlotRiboDensityAroundTriAAMotifs -i PPP_motifDensity_dataframe.txt -o PPP -g si-Ctrl,si-eIF5A -r si-Ctrl-1,si-Ctrl-2__si-eIF5A-1,si-eIF5A-2 --mode mean --ymax 0.2*

We have known that ribosome density at proline increased on almost 80% in si-eIF5A samples compared with si-Ctrl samples [(Figure 3C)](http://static.zybuluo.com/sherking/rl0wsq9zbrgnjg5f8alz00p5/AADensity.png). Now if we want to explore the ribosome density around the P/E site of poly-proline, we could use *RiboDensityAroundTripleteAAMotifs* and *PlotRiboDensityAroundTriAAMotifs* to achieve it. And the results showed that compared with positions around the P/E site of poly-proline, ribosome density on poly-proline were increased in si-eIF5A samples as well as ribosome density on poly-Aspartic acid[(Figure 4A)](http://static.zybuluo.com/sherking/soy2p4k58enaxjo32cvgmglw/triAADensity.png). In addition, we also found that ribosome density on poly-Lysine were decreased which have never been reported yet [(Figure 4B)](http://static.zybuluo.com/sherking/soy2p4k58enaxjo32cvgmglw/triAADensity.png).

- **As for a tri-AA motifs list which contains part of information like this:**

*motifs
PPP
DDD
SPP
LPP*

Using following command to do such job:

*RiboDensityAroundTripleteAAMotifs -f <attributes.txt> -c <longest.transcripts.info.txt> -o <output_prefix> -M RPKM -S <select_trans.txt> -l 100 -n 10 --table 1 -F <longest_cds_sequence.fa> --motifList1 tri_AA_motifs1.txt --motifList2 tri_AA_motifs2.txt

PlotRiboDensityAroundTriAAMotifs -i tri_AA_motifDensity_dataframe.txt -o output_prefix -g si-Ctrl,si-eIF5A -r si-Ctrl-1,si-Ctrl-2__si-eIF5A-1,si-eIF5A-2 --mode mean --ymax 0.2*

This step would generate ribosome density plots on each tri-AA motif in *tri_AA_motifs1.txt* and *tri_AA_motifs2.txt* as well as a total ribosome density for tri-AA motifs in each tri-AA motif lists.

- **Pausing score of each triplete amino acid.**

*## pausing score calculation
PausingScore -f <attributes.txt> -c <longest.transcripts.info.txt> -o <output_prefix> -M RPKM -S <select_trans.txt> -l 100 -n 10 --table 1 -F <longest_cds_sequence.fa>

## process pausing score
ProcessPausingScore -i si-Ctrl-1_pausing_score.txt,si-Ctrl-2_pausing_score.txt,si-eIF5A-1_pausing_score.txt,si-eIF5A-2_pausing_score.txt -o <output_prefix> -g si-Ctrl,si-eIF5A -r si-Ctrl-1,si-Ctrl-2__si-eIF5A-1,si-eIF5A-2 --mode raw --ratio_filter 2 --pausing_score_filter 10*

This step would generate a pausing score file as for each sample. In order to pick those tri-AA motifs enriched more ribosomes out, we offer *ProcessPausingScore* to do such job and output an **position weight matrix** for E,P,A sites of a tri-AA motif which later on could be used for motif logo plot using [**Seq2Logo**](http://www.cbs.dtu.dk/biotools/Seq2Logo/).

As for parameters in *ProcessPausingScore*, *-i* is the pausing score file we need to input, and all pausing score file should be separated by comma. *–ratio_filter 2* means the FC(treat/ctrl)>=2 and *–pausing_score_filter 10* means pausing score in si-eIF5A group must be larger than 10. *–mode raw* means input is the raw pausing score generated by *PausingScore*; *–mode ratio* means input is the processed file with this format

*motif si-Ctrl si-eIF5A ratio
PPP 9.583728008472916 32.40390126709601 3.3811374069097035
DDD 7.864248366516 18.973658903418116 2.4126474672650478
SPP 5.095062641097612 14.244816613232201 2.7958079451921103
LPP 6.269550858040044 13.567581067294325 2.1640435454631204
DDP 3.533105599301455 13.297017644747527 3.763549452746766
APP 3.8178063397299846 11.921477030003814 3.1225986781841226*

And the last **position weight matrix** looks like this:

*A D L P S
0 0.14909086080045236 0.4526622484437558 0.10332391327033515 0.16143499003013836 0.13348798745531829
1 0.0 0.45266224844375585 0.0 0.5473377515562442 0.0
2 0.0 0.1367730692993923 0.0 0.8632269307006076 0.0*

Where the *0,1,2* represent the E,P,A site of a tri-AA motif. Using [**Seq2Logo**](http://www.cbs.dtu.dk/biotools/Seq2Logo/) for logo plot and the final results were showed on [(Figure 4C)](http://static.zybuluo.com/sherking/soy2p4k58enaxjo32cvgmglw/triAADensity.png). As we could see that after the expression of eIF5A was interfered, ribosome density on poly-proline and poly-Aspartic acid are much increased.

*## this tool needs a python2.7 environment
PATH=/tools/python2:$PATH
Seq2Logo.py -f <pwm.txt> -u probability -I 5 -o <output_preifx> --format PDF*


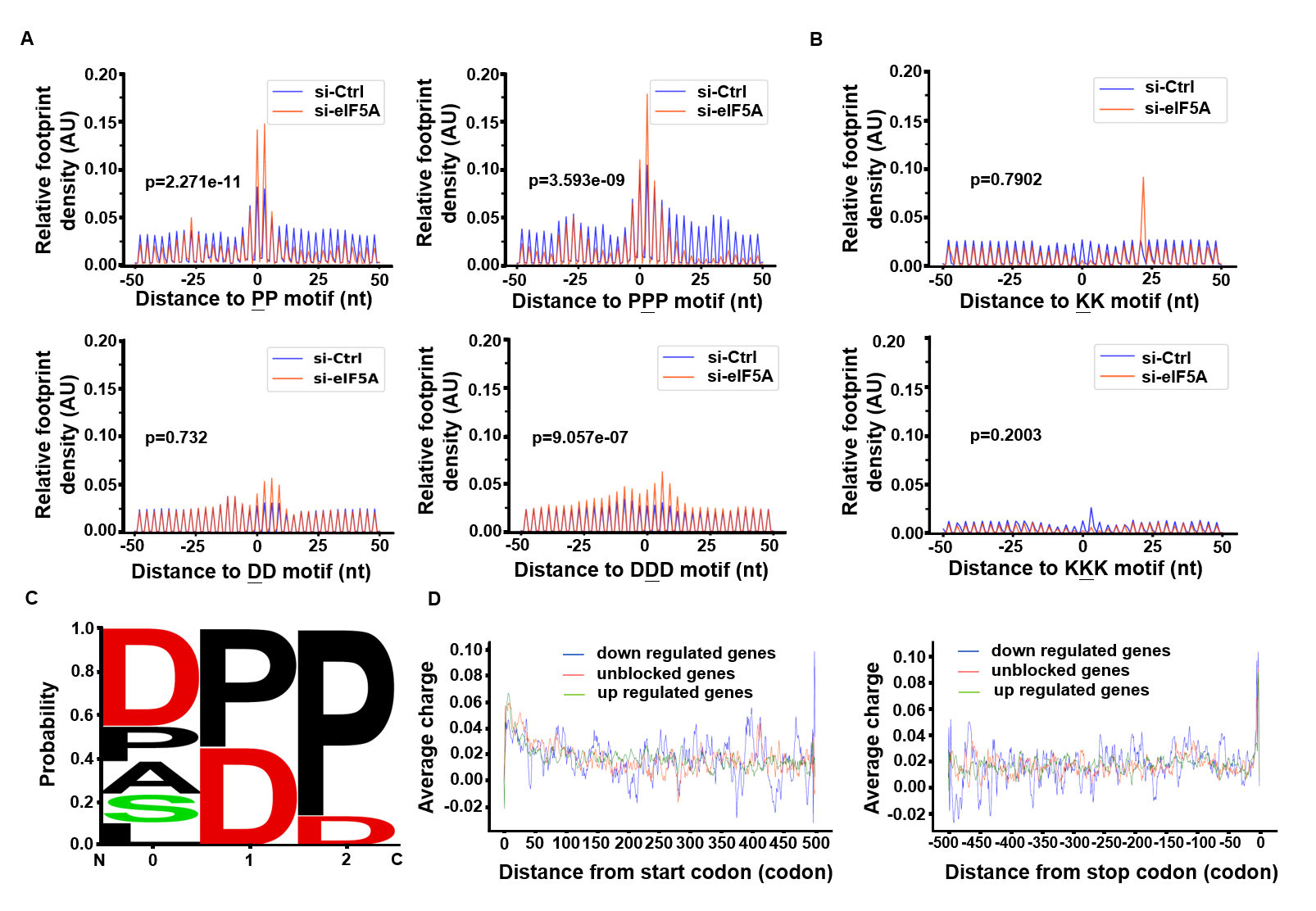


**Figure 4: Ribosome density among tri-AA motifs**. (**A).** Relative ribosome density on poly-proline and poly-Aspartic acid. (**B).** Relative ribosome density on poly-Lysine. (**C)**. The tri-AA motifs with enriched ribosome, reported by RiboMiner. 0,1,2 represents E, P, A site of a tri-AA motif, respectively. (**D).** Average charge of each position along transcripts. All these analyses above were based on the 2954 up-regulated transcripts.

- **RPFdist calculation.**

*RPFdist -f <attributes.txt> -c <longest.transcripts.info.txt> -o <output_prefix> -M RPKM -S <select_trans.txt> -l 100 -n 10 -m 1 -e 5*

- **Local tRNA adaptation index and global tRNA adaptation index**

*## tAI calculation
tAI -i 2954_up_cds_sequences.fa,1598_unblocked_cds_sequences.fa,433_down_cds_sequences.fa -N tRNA_GCNs_Saccharomyces_cerevisiae.txt -o test -u 0 -d 500 -t 2954_up,1598_unblocked,433_down
## tAI plot
tAIPlot -i <output_prefix_tAI_dataframe.txt> -o <output_prefix> -u 0 -d 500 --mode all --start 5 --window 7 --step 1*

- **Local codon adaptation index and global codon adaptation index**

*## cAI calculation
cAI -i $workdir/2954_up_cds_sequences.fa,$workdir/1598_unblocked_cds_sequences.fa,$workdir/433_down_cds_sequences.fa -o $workdir/featureAnalysis/test.yeast -u 0 -d 500 -t 2954_up,1598_unblocked,433_down --reference reference.fa
## cAI plot
cAIPlot -i <output_prefix_local_cAI_dataframe.txt> -o <output_prefix> -u 0 -d 500 --mode all --start 5 --window 7 --step 1*

- **Hydrophobicity calculation and Charge amino acids**

*## hydrophathy calculation
hydropathyCharge -i $workdir/2954_up_cds_sequences.fa,$workdir/1598_unblocked_cds_sequences.fa,$workdir/433_down_cds_sequences.fa -t 2954_up,1598_unblocked,433_down -o $workdir/featureAnalysis/test.yeast_hydropathy -u 0 -d 500 --index $workdir/featureAnalysis/hydropathy.txt
## charge calculation
hydropathyCharge -i $workdir/2954_up_cds_sequences.fa,$workdir/1598_unblocked_cds_sequences.fa,$workdir/433_down_cds_sequences.fa -t 2954_up,1598_unblocked,433_down -o $workdir/featureAnalysis/test.yeast_charge -u 0 -d 500 --index $workdir/featureAnalysis/AA_charge.txt*

the both index file looks like:

*## hydrophobicity index download from AAindex
AA amio_acids hydropathy
A Ala 1.8
R Arg -4.5
N Asn -3.5
D Asp -3.5
C Cys 2.5
...
## charge index
AA amio_acids charge
A Ala 0
R Arg 1
N Asn 0
D Asp -1
C Cys 0
...*

among which the hydrophobicity index are downloaded from [AAindex](https://www.genome.jp/aaindex/). Later on, use *PlotHydropathyCharge* for plot:

*PlotHydropathyCharge -i test.yeast_hydropathy_values_dataframe.txt -o test_hydropathy -u 0 -d 500 --mode all --ylab "Average Hydrophobicity"
PlotHydropathyCharge -i test.yeast_charge_values_dataframe.txt -o test_charge -u 0 -d 500 --mode all --ylab "Average Charge"*

In the [original paper](https://www.ncbi.nlm.nih.gov/pubmed/28392174), the authors reported that the eIF5A alleviates stalling on many motifs besides polyproline tracts, but did not explain the exact reason why ribosomes move slowly on the first 100 codons along transcripts. We tried a lot to explain this but still failed. However, by calculating the tRNA adaptation index (tAI) and codon adaptation index (cAI), we found those transcripts enriched more ribosomes tend to have much smaller tAI and cAI values compared wtih other transcripts whatever as for local tAI [(Figure 5B)](http://static.zybuluo.com/sherking/r877xvlk0zyf0q6mfelxzbiv/CAItAI.png), local cAI [(Figure 5A)](http://static.zybuluo.com/sherking/r877xvlk0zyf0q6mfelxzbiv/CAItAI.png) or the global tAI or cAI [(Figure 5D)](http://static.zybuluo.com/sherking/r877xvlk0zyf0q6mfelxzbiv/CAItAI.png). Further, we found those transcripts enriched more ribosomes tend to have much smaller hydrophobicity [(Figure 5C)](http://static.zybuluo.com/sherking/r877xvlk0zyf0q6mfelxzbiv/CAItAI.png) and there is almost no difference among different gene sets for charges [(Figure 4D)](http://static.zybuluo.com/sherking/soy2p4k58enaxjo32cvgmglw/triAADensity.png).


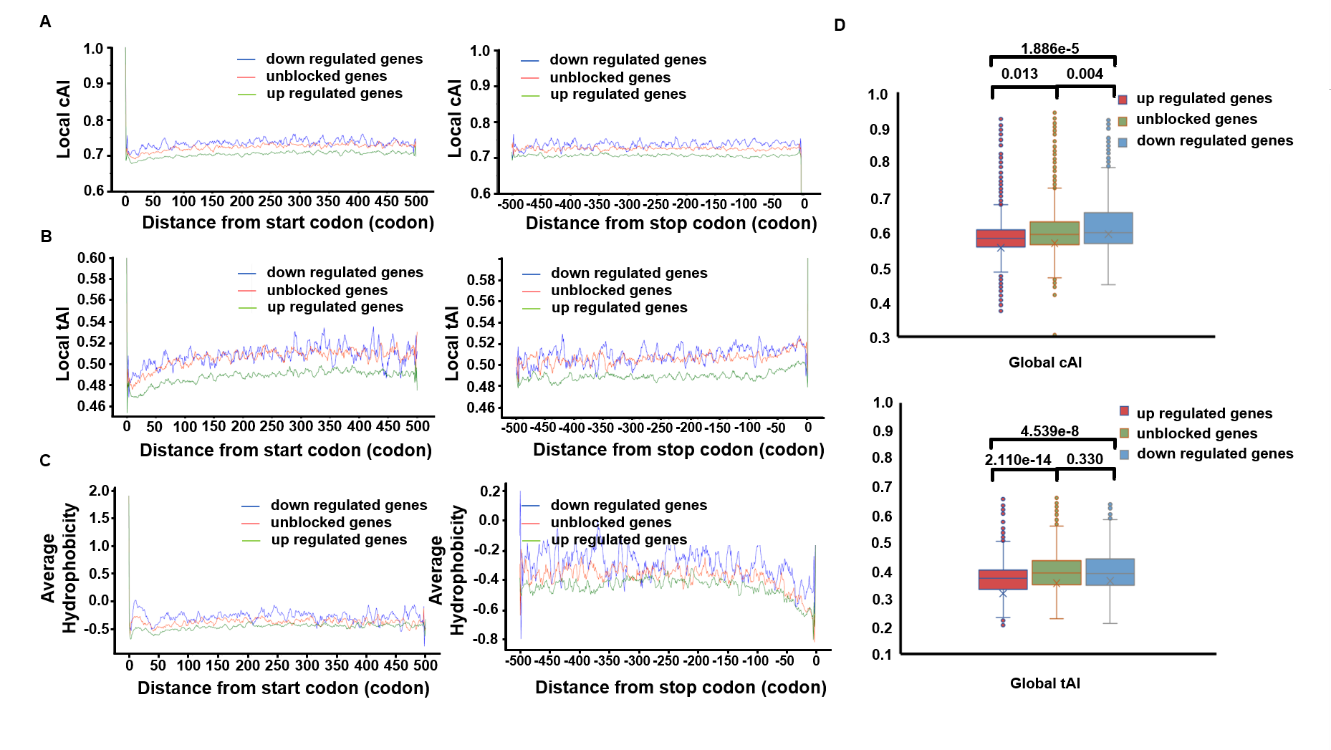


**Figure 5: Results of Feature Analysis (FA) by RiboMiner**. (**A)**. Distribution of local cAI along transcripts for different gene sets. (**B)**. Distribution of local tAI along transcripts for different gene sets. (**C)**. Average hydrophobicity of each position along transcripts for different gene sets. (**D)**. Distribution of global cAI and global tAI for different gene sets. p-values with t.test: Global cAI, 0.012915237 for up-regulated genes vs unblocked gene, 1.88585e-05 for up-regulated genes vs down regulated genes; Global tAI: 2.11048e-14 for up-regulated genes vs unblocked genes, 4.53906e-08 for up-regulated genes vs down regulated genes.

## Enrichment Analysis (EA)

The enrichment analysis is used for identifying potential co-translation events just like [Ayala Shiber, et al](https://www.nature.com/articles/s41586-018-0462-y) did. Therefore, there are two input files for this step. One is the total translatome file and the other is the IP translatome file. Here we used [**GSE116570**](https://www.ncbi.nlm.nih.gov/geo/query/acc.cgi?acc=GSE116570) datasets to show the useage of **RiboMiner**. The enrichment analysis contains four steps:

- **Step 1: Calculate ribosome density at each position for each transcript.**

*RiboDensityAtEachPosition -c <longest.transcripts.info.txt> -f <attributes.txt> -o <output_prefix> -U codon*

where *attributes.txt* and *longest.transcripts.info.txt* are files we have talked above. This step would generate two files for each sample. One is the ribosome density file which would be used for following analysis. The Other is the coverage file, containing coverage of each transcript on CDS region.

- **Step 2: Calculate mean ribosome density for different replicates.**

*## use IP:MES1 for example
enrichmentMeanDensity -i MES1-IP-1_cds_codon_density.txt,MES1-IP-2_cds_codon_density.txt -o MES1_mean*

*-i* represents ribosome density files of different replicates generated by *RiboDensityAtEachPosition*, which should be separated by comma. This would generate a mean density file for each sample like *output_prefix_mean_density.txt*, used for enrichment analysis. If there is no replicates, just pass this step.

- **Step 3: Enrichment analysis.**

*## all transcripts
EnrichmentAnalysis --ctrl MES1_total_mean_density.txt --treat MES1_IP_mean_density.txt -c <longest.transcripts.info.txt> -o <output_prefix> -U codon -M RPKM -l 150 -n 10 -m 1 -e 30 --CI 0.95 -u 0 -d 500
## specific transcripts
EnrichmentAnalysis --ctrl MES1_total_mean_density.txt --treat MES1_IP_mean_density.txt -c <longest.transcripts.info.txt> -o ARC1 -U codon -M RPKM -l 150 -n 10 -m 1 -e 30 --CI 0.95 -u 0 -d 500 -S ARC1.txt --id-type transcript-id*

where *ARC1.txt* contains:

*trans_id
YGL105W*

- **Step 4: Plot the enrichment ratio.**

*PlotEnrichmentRatio -i ARC1_enrichment_dataframe.txt -o <output_prefix> -u 0 -d 500 --unit codon --mode all --slide-window y --axhline 1*

- **Notes: if you want to see the enrichment ratio for a single transcript, the EnrichmentAnalysisForSingleTrans would be helpful.**

*EnrichmentAnalysisForSingleTrans -i all_codon_ratio.txt.txt -s ARC1 -o ARC1 -c <longest.trans.info.txt> --id-type gene_name --slide-window y --axhline 1*

*
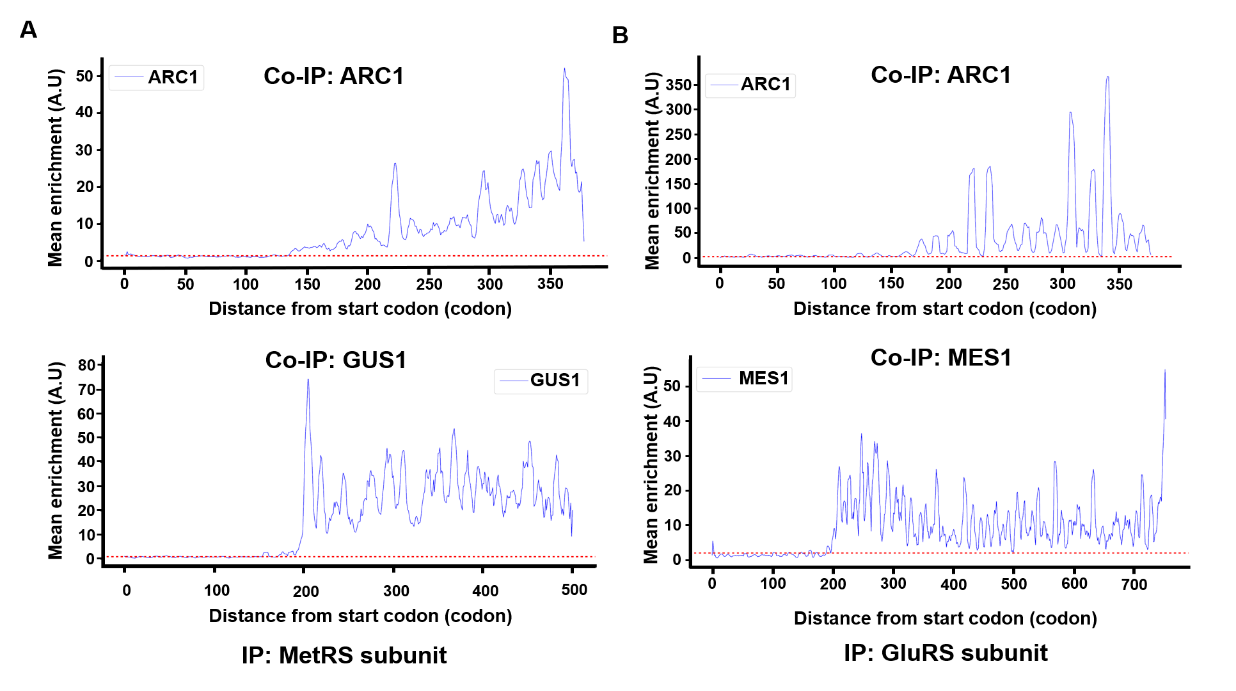
*

**Figure 6: The results of Enrichment Analysis by RiboMiner**. (**A)**. Engagement of nascent ARC1 (top), GUS1 (bottom) by C-terminally tagged MetRS. (**B)**. Engagement of nascent ARC1 (top), MES1 (bottom) by C-terminally tagged GluRS. The red dotted lines represent two-fold threshold.

# Methods

## Pre-processing of the ribosome profiling data

The yeast reference genome assembly (Saccharomyces cerevisiae.R64-1-1) and the annotation file downloaded from the [**Ensembl genome browser**](http://www.ensembl.org/index.html?redirect=no) were used for all analyses. The pre-processing procedure of the ribosome profiling has been described previously [(Xiao et al., 2016)](https://www.nature.com/articles/ncomms11194). [**FastQC**](http://www.bioinformatics.babraham.ac.uk/projects/fastqc/) was used for quality control and the adaptor sequence (CTGTAGGCACCATCAAT) in the raw reads of ribosome profiling was trimmed using the [**cutadapt**](https://cutadapt.readthedocs.io/en/stable/) [(Martin, 2011)](http://journal.embnet.org/index.php/embnetjournal/article/view/200/479). Reads with length between 25-35 nt were used for ribosome profiling analyses. Low-quality reads with Phred quality scores lower than 25 (>75% of bases) were removed using the [**fastx quality filter**](http://hannonlab.cshl.edu/fastx_toolkit/). Then sequence reads originating from rRNAs were identified and discarded by aligning the reads to yeast non-coding sequences downloaded from [**Ensembl genome browser**](http://www.ensembl.org/index.html?redirect=no) using [**Bowtie 1.1.2**](http://bowtie-bio.sourceforge.net/index.shtml) [(Langmead et al., 2009)](https://genomebiology.biomedcentral.com/articles/10.1186/gb-2009-10-3-r25) with no mismatch allowed. The remaining reads were mapped to the genome using [**STAR**](http://labshare.cshl.edu/shares/gingeraslab/www-data/dobin/STAR/STAR.posix/doc/STARmanual.pdf) [(Dobin et al., 2013)](https://www.ncbi.nlm.nih.gov/pmc/articles/PMC3530905/) with the following parameters: –runThreadN 8 –outFilterType Normal –outWigType wiggle –outWigStrand Stranded –outWigNorm RPM –alignEndsType EndToEnd –outFilterMismatchNmax 1 –outFilterMultimapNmax 1 –outSAMtype BAM SortedByCoordinate –quantMode TranscriptomeSAM GeneCounts –outSAMattributes All.

## Quality control of the ribosome profiling data

Thanks to its sub-codon resolution, ribosome profiling reveals the precise locations of the peptidyl-site (P-site) of the 80S ribosome in the RPF reads. 3-nt periodicity of the RPF reads aligned by their P-sites was used as a strong evidence of activate translation [(Ingolia et al., 2009)](https://www.ncbi.nlm.nih.gov/pubmed/19213877). Here we used the metaplots from [**RiboCode**](https://github.com/xryanglab/RiboCode) [(Xiao et al., 2018)](https://academic.oup.com/nar/article/46/10/e61/4925760) and [*Periodicity*](https://github.com/xryanglab/RiboMiner/blob/master/RiboMiner/Periodicity.py) from [**RiboMiner**](https://github.com/xryanglab/RiboMiner) to assess the 3-nt periodicity of all Ribo-seq samples. Read length distribution was done by [*LengthDistribution*](https://github.com/xryanglab/RiboMiner/blob/master/RiboMiner/LengthDistribution.py) and DNA contamination was checked by [*StatisticReadsOnDNAsContam*](https://github.com/xryanglab/RiboMiner/blob/master/RiboMiner/StatisticReadsOnDNAsContam.py).

## Metagene analyses of the ribosome profiling data

The read counts at each codon of the transcripts were calculated using [*MetageneAnalysis*](https://github.com/xryanglab/RiboMiner/blob/master/RiboMiner/MetageneAnalysis.py). As for each transcript, the read counts vector is written as:

$$read\_counts_{trans_{i}}=[c_{1},c_{2},...,c_{n-1},c_{n}] (1)$$

Where $c_{i}$ is the read count at codon position $i$ in the transcript, and $n$ represents the length of the ORF in unit of codon. The read counts at each position were normalized by the mean value of each read counts vector without the first several codons (e.g.30) to exclude the accumulated reads around the start codon. For genes with multiple transcript isoforms, only the longest isoform was used when parsing the genome annotation files. For the metagene analyses at the global scale, the transcripts with more than 100 codons were used. After the filtering steps above, all transcripts were lined up and the mean read density at each position was be calculated as:

$$\overset{⃗}{read\_density}=\frac{\sum_{N} c_{i}}{N} (2)$$

Where $N$ is the numbers of transcripts retained, and $c_{i}$ represents the read density at codon $i$ position. Finally, all mean read density at each position formed a vector that was then used for metagene plots. As for metagene analysis for the whole transcript region, the coverage profiles were calculated independently for 5’UTR, CDS and 3’UTR by sampling the normalized signal in 15, 90, 15 bins, respectively. The bin numbers were set by users. Normalized transcripts were averaged together in a vectored way to plot the coverage distribution.

## Polarity calculation

To quantify the differences in the position of ribosomes along transcripts, we computed a polarity score for every gene just as [**Anthony P, et al**](https://www.ncbi.nlm.nih.gov/pubmed/28392174) described before [(Schuller et al., 2017)](https://www.ncbi.nlm.nih.gov/pubmed/28392174). The polarity at position $i$ in a gene of length $l$ is defined as follows:

$$p_{i}=\frac{d_{i}w_{i}}{\sum_{i=1}^{l} d_{i}} (3)$$

Where

$$w_{i}=\frac{2i-(l+1)}{l-1} (4)$$

The $d_{i}$ and $w_{i}$ represent the ribosome density and normalized distance from the center of a gene at position $i$ respectively. Polarity score for a gene is the total sum of $p_{i}$ at each position. Also, we excluded the first and the last 15 nt of coding sequences from our analyses. Genes with more than 64 reads in coding sequences were used for generating the polarity plots.

## Read density at each codon and amino acid

Read density at each codon or amino acid along a transcript was calculated, after which densities covered on each kind of codon or amino acid among all transcripts were summed up separately. And then, density change at each kind of codon or amino acid was calculated between two different conditions.

## Hydrophobicity index

Hydrophobicity index (also called hydropathy index) of an amino acid is a number representing the hydrophobic or hydrophilic properties of its sidechain [(Kyte and Doolittle, 1982)](https://www.ncbi.nlm.nih.gov/pubmed/7108955). The hydrophobicity index was download from [**AAindex**](https://www.genome.jp/aaindex/) and the mean values at each position along the amino acid sequences were feed into a vector used for metagene analysis.

## Charges of amino acid

The number 1, -1 and 0 were assigned for amino acids with positive charges (Lys and Arg), negative charges (Asp and Glu) and those without any charges, respectively. As for each amino acid chain selected, charge values at each position were computed and averaged after all amino acid chains lined up, which would be used for plot.

## The local tAI and global tAI

The local tAI (tRNA adaptation index) was computed by a procedure as previously described [(Tuller et al., 2010)](https://www.ncbi.nlm.nih.gov/pubmed/20403328). That is, let $n_{i}$ be the number of tRNA isoacceptors recognizing codon $i$. Let $tGCN_{ij}$ be the copy number of the $j$th tRNA that recognizes the $i$th codon, and let $S_{ij}$ be the selective constraint on the efficiency of the codon-anticodon coupling. The absolute adaptiveness, $W_{i}$, for each codon $i$ as

$$W_{i}=\sum_{j=1}^{n_{i}} (1-S_{ij})tGCN_{ij} (5)$$

From the absolute adaptiveness we could get the relative adaptiveness value of codon $i$, namely $w_{i}$, by normalizing the $W_{i}$’s values (dividing them by the maximal of all $W_{i}$).

$$w_{i}=\frac{W_{i}}{maxW_{i}} (6)$$

The global tAI of a gene later would be calculated as the geometric mean of $w_{i}$. However, we also intended to observe the tAI values at each position along transcripts. Therefore, $w_{i}$ would be treated as the local tRNA adaptiveness index for codon $i$, after which all local tAI values of each transcript would be lined up and mean values at each position would be computed used for plot. The copy numbers of each tRNA were download from [**GtRNAdb**](http://gtrnadb.ucsc.edu/) and the scores $S_{ij}$ for wobble nucleoside-nucleoside paring were from the results of Tamir Tuller’s work [(Tuller et al., 2010)](https://www.ncbi.nlm.nih.gov/pubmed/20403328).

## The local cAI and the global cAI

The method for computing the local cAI (codon adaptation index) is similar to the procedure for local tAI. The global cAI was first defined by [**Sharp and Li**](https://www.researchgate.net/profile/Paul_Sharp2/publication/19615259_The_codon_Adaptation_Index-A_measure_of_directional_synonymous_codon_usage_bias_and_its_potential_applications/links/53d66b290cf2a7fbb2eaa3e0/The-codon-Adaptation-Index-A-measure-of-directional-synonymous-codon-usage-bias-and-its-potential-applications.pdf) [(M.Sharpl and Li, 1987)](https://www.researchgate.net/profile/Paul_Sharp2/publication/19615259_The_codon_Adaptation_Index-A_measure_of_directional_synonymous_codon_usage_bias_and_its_potential_applications/links/53d66b290cf2a7fbb2eaa3e0/The-codon-Adaptation-Index-A-measure-of-directional-synonymous-codon-usage-bias-and-its-potential-applications.pdf) associated a value in [0,1] as

$$cAI(g)=(\prod_{k=1}^{L} w_{k})^{1/L} (7)$$

where cAI is the codon adaptation index of gene $g$, $L$ is the number of codons in the gene, and $w_{k}$ is the weight of the $k$th codon in the gene sequence [(Carbone et al., 2003)](https://academic.oup.com/bioinformatics/article/19/16/2005/241992) which was defined as

$$w_{ij}=\frac{x_{ij}}{y_{j}} (8)$$

where $x_{ij}$ is the frequency of codon $i$ for the amino acid $j$ occurs in a set of gene sequences $S$. $y_{j}$ is the maximal frequency of its sibling codons. Genes with cAI values close to 1 are made by highly frequent codons [(Carbone et al., 2003)](https://academic.oup.com/bioinformatics/article/19/16/2005/241992). The weight $w_{ij}$ was defined as the local cAI in our research.

## Enrichment analysis

Read counts along each transcript were computed as described in the section above. Moving-average method was used to obtain read density at each position along a transcript (size of the window: 7 codons; step size: 1 codon). Enrichment ratios were calculated at each position. Remaining zeros at some positions were replaced with the number 1. Only transcripts longer than 100 codons with RPKM in the coding region larger than 10 were used. Meta-analysis for the enrichment ratios was done like the same way as metagene analysis.

# References

Schuller, A.P., Wu, C.C., Dever, T.E., Buskirk, A.R., and Green, R. (2017). eIF5A Functions Globally in Translation Elongation and Termination. Mol Cell 66, 194-205 e195.

Shiber, A., Doring, K., Friedrich, U., Klann, K., Merker, D., Zedan, M., Tippmann, F., Kramer, G., and Bukau, B. (2018). Cotranslational assembly of protein complexes in eukaryotes revealed by ribosome profiling. Nature 561, 268-272.

Xiao, Z., Zou, Q., Liu, Y., and Yang, X. (2016). Genome-wide assessment of differential translations with ribosome profiling data. Nat Commun 7, 11194.

Martin, M. (2011). Cutadapt removes adapter sequences from high-throughput sequencing reads. 2011 17, 3 %J EMBnet.journal.

Langmead, B., Trapnell, C., Pop, M., and Salzberg, S.L. (2009). Ultrafast and memory-efficient alignment of short DNA sequences to the human genome. Genome Biology 10.

Dobin, A., Davis, C.A., Schlesinger, F., Drenkow, J., Zaleski, C., Jha, S., Batut, P., Chaisson, M., and Gingeras, T.R. (2013). STAR: ultrafast universal RNA-seq aligner. Bioinformatics 29, 15-21.

Ingolia, N.T., Ghaemmaghami, S., Newman, J.R., and Weissman, J.S. (2009). Genome-wide analysis in vivo of translation with nucleotide resolution using ribosome profiling. Science 324, 218-223.

Xiao, Z., Huang, R., Xing, X., Chen, Y., Deng, H., and Yang, X. (2018). De novo annotation and characterization of the translatome with ribosome profiling data. Nucleic Acids Res 46, e61.

Kyte, J., and Doolittle, R.F. (1982). A Simple Method for Displaying the Hydropathic Character of a Protein. J Mol Biol 157, 105-132.

Tuller, T., Carmi, A., Vestsigian, K., Navon, S., Dorfan, Y., Zaborske, J., Pan, T., Dahan, O., Furman, I., and Pilpel, Y. (2010). An evolutionarily conserved mechanism for controlling the efficiency of protein translation. Cell 141, 344-354.

M.Sharpl, P., and Li, W.-H. (1987). The codon adaptation index-a measure of directional synonymous codon usage bias, and its potential applicaitons. Nucleic Acids Research 15.

Carbone, A., Zinovyev, A., and Kepes, F. (2003). Codon adaptation index as a measure of dominating codon bias. Bioinformatics 19, 2005-2015.

Lee, B. D. (2018). Python Implementation of Codon Adaptation Index. Journal of Open Source Software, 3 (30), 905
